# Dynamic person-position matching decision method based on hesitant fuzzy number information

**DOI:** 10.1038/s41598-024-54177-8

**Published:** 2024-02-15

**Authors:** Qi Yue, Liezhang Liu, Yuan Tao

**Affiliations:** School of Management, Shanghai University of Engineering Science, Shanghai, 201620 China

**Keywords:** Dynamic person-position matching, Stable matching, Hesitant fuzzy number, Dynamic satisfaction, Stable person-position matching model, Engineering, Mathematics and computing

## Abstract

In view of the fact that people pay more and more attention to the principle of "getting the position according to the person" and "adapting the person to the position" in person-position matching, a dynamic person-position matching decision method based on hesitant fuzzy numbers is proposed. First, the dynamic person-position matching problem with hesitant fuzzy numbers is described. Then, according to hesitant fuzzy evaluation matrices of positions and candidates, expected score matrices of bilateral subjects are calculated. Furthermore, based on the idea of the generalized optimal order method and the dominant correlation and the missing correlation coefficients, satisfaction means of people and positions are calculated. According to satisfaction means, growth satisfactions at each period are obtained, and then the exponential decay formula is used to determine weights of growth satisfactions. Dynamic satisfactions of bilateral subjects are calculated by aggregating initial satisfaction means and growth satisfactions. On this basis, a stable person-position matching model considering dynamic satisfactions is established and then is solved to obtain the optimal stable person-position matching scheme. Finally, the feasibility and effectiveness of the proposed method are verified by an example analysis of person-position matching. Main contributions of this paper are as follows: an effective calculation method for the missing correlation coefficient is presented; a novel effective calculation method for dynamic satisfactions is proposed by introducing the correlation parameter to combine the missing correlation coefficient with the dominant correlation coefficient; an effective stable person-position matching model considering dynamic satisfactions is established.

## Introduction

Decision is a complex process in which people ultimately determine what actions to take to achieve specific goals through a series of thinking processes and volitional actions in the face of different choices. This process involves the collection and processing of information, as well as the analysis and calculation of possible influencing factors to make the best choice. With the development of society, decision theory has been enriched, and many scholars have carried out optimization research. To improve the prediction accuracy in complex systems, Wang et al.^1^ proposed an optimization method combining fuzzy K-clustering and fuzzy neural network; Tešić et.al.^2^ applied the DIBR II-BM-CoSo MCDM model to the optimal selection of military combat assault boats. Zhao et.al.^3^ observed multi-objective integrated optimization results of predictive maintenance and production scheduling from the perspective of delivery cycle constraints. As an important tool for decision, fuzzy set theory is also enriched, and related research is constantly emerging. For example, Jana et.al.^4^ proposed an interval-valued image fuzzy uncertain linguistic dombi operator and applied it to the selection of industrial funds; Riaz et.al.^5^ improved the efficiency of green supply chain by a linear Diophantine fuzzy soft-max aggregation operator; Khan et.al.^6^ proposed a Fematean fuzzy set algorithm of generalized and group generalized parameters, and applied it to decision making problems; Abid et.al.^7^ used edge cloud computing and deep learning tools to assess risks in China 's international trade and investment.

After the end of the epidemic, the situation of internal competition in various industries is severe. To save costs and improve core competitiveness, many enterprises optimize the allocation of people and positions. In such a situation, people become more cautious about their career choices. At the same time, companies are also more stringent in recruiting candidates. The relationship between people and positions is no longer a short-term matching relationship, but pays more and more attention to the principle of " the person in the position " and " the person is suitable for the position ". Therefore, it is quite important to find an efficient person-position matching scheme.

Bilateral matching theory has attracted much attention and is widely used in many fields. For example, Gale and Shapley^8^ studied the college admission and stable marriage matching in 1962. Miao et al.^9^ studied a bilateral matching model between cross-border demanders and suppliers. Zhao et al.^10^ studied a stable bilateral satisfactory matching problem of carpooling system based on preference orders. Han et al.^11^ proposed a bilateral matching model of service providers and demanders considering peer effects and synergies. Jiang et al.^12^ proposed a bilateral matching decision model for complex product systems based on life cycle sustainability, aiming at the problem that different matching schemes of manufacturers and service providers affect the continuous use of complex product systems. In addition, the person-position matching problem, as one of the most typical problems in the field of bilateral matching, has also attracted much attention of scholars. For example, Dai et al.^13^ used BP neural network to study the problem of person-position matching, and constructed a person-position matching evaluation model based on BP neural network. Wang et al.^14^ constructed an end-to-end person-position matching model and conducted matching experiments with actual recruitment data, which provided some clues for future person-position matching work. Beatriz^15^ studied a many-to-one stable matching problem between companies and unemployed workers. Liu et al.^16^ applied the multi-criteria bilateral matching method of TODIM technology to person-position matching. Yu et al.^17^ designed an intuitionistic fuzzy bilateral matching model to solve the person-position matching problem. Liang et al.^18^ proposed an interval-valued intuitionistic fuzzy bilateral matching model considering the level of automation to solve the human–machine position matching in intelligent production lines. Yang et al.^19^ proposed a new hybrid bilateral matching method based on I-BTM and LSGDM suitable for overseas high-level talents and position matching problems.

The above research has greatly enriched the scope and perspective of bilateral matching decision field. However, with the popularity of internet technology, the decision preference of bilateral subjects is no longer the single preference ranking information, but more dynamic information that changes over time. For example, Wang et al.^20^ studied the stable matching of dynamic ride-sharing systems. Zhao et al.^21^ proposed a dynamic bilateral matching decision method. Liang et al.^22^ proposed a multi-attribute dynamic bilateral matching method for talent sharing market under an incomplete preference order environment. Li et al.^23^ discussed the problem of dynamic resource allocation on multi-category bilateral platforms. Zhao et al.^24^ proposed an online doctor-patient dynamic stable matching model based on the online doctor-patient matching problem with incomplete information.

The above literatures have expanded the application of person-position matching and dynamic matching, but there are still some shortcomings. On the one hand, due to the complexity of practical problems and the cognition limitation of bilateral subjects, bilateral subjects often give preferences in the form of hesitant fuzzy numbers. However, most of the existing dynamic matching studies start from preference orders, which has great limitations in actual research process. On the other hand, there are few studies on person-position matching based on hesitant fuzzy numbers and dynamic person-position matching. Therefore, this paper proposes a dynamic person-position matching decision method from the perspective of stable matching under a hesitant fuzzy environment, and the method proposed in this paper is compared with the above related literatures, as shown in Table 1.

**Table 1:** Comparative analysis of the above literatures with this paper.

Author	Fuzzy information	Dynamic information	Stable matching	Model
Dai and Hu	No	No	No	No
Wang et al.	No	No	No	Yes
Beatriz	No	No	Yes	No
Liu and Wang	Intuitionistic linguistic number	No	No	Yes
Yu and Xu	Intuitionistic fuzzy set	No	No	Yes
Liang et al.	Interval-valued intuitionistic fuzzy set	No	No	Yes
Yang et al.	Hesitant fuzzy language	No	No	Yes
Wang et al.	No	Yes	Yes	No
Liang et al.	No	Yes	No	No
Li et al.	No	Yes	No	Yes
Zhao et.al.	Probabilistic language information entropy	Yes	Yes	Yes
This paper	Hesitant fuzzy set	Yes	Yes	Yes

Research motivations of this study are as follows:

1. It is a novel idea to apply hesitant fuzzy set theory to person-position matching. This study enriches person-position matching decision.
2. Satisfactions of bilateral subjects at each stage are very important, and the missing correlation coefficient needs to be introduced to calculate satisfactions of bilateral subjects.
3. In the actual enterprise promotion process, satisfactions of bilateral subjects are constantly changing in different stages. Therefore, dynamic satisfactions need to be calculated by combining initial satisfactions and growth satisfactions.
4. The stable person-position matching model under a hesitant fuzzy environment can reveal the uncertainty and fuzziness of matching decision process more flexibly and in more detail. Hence, it is necessary to establish a person-position matching decision model in a hesitant fuzzy environment.

Main contributions of this paper are as follows:

1. A dynamic person-position matching decision method based on hesitant fuzzy numbers is proposed.
2. The missing correlation coefficient is proposed to provide a basis for the calculation of satisfactions of bilateral subjects.
3. A novel calculation method for initial satisfactions of bilateral subjects that combines the dominant and missing correlation coefficients is designed.
4. Growth satisfactions are calculated to facilitate bilateral subjects to understand the status of each stage and improve their behaviors in time.
5. A stable person-position matching model considering dynamic satisfactions is established, which provides a more reasonable solution to the problem of person-position matching in the promotion process.

The remaining structure of this paper is as follows: “Preparatory knowledge” section introduces concepts of hesitant fuzzy numbers and stable person-position matching. “Dynamic person-position matching decision under a hesitant fuzzy environment” section discusses the dynamic person-position matching decision under a hesitant fuzzy environment. “Example analysis” section verifies the effectiveness and feasibility of the proposed method through a case of person-position matching. “Conclusions” section summarizes this paper.

## Preparatory knowledge

### Hesitant fuzzy number

#### Definition 1 (Ref.^25^)

Let $X = \{x_{1} ,x_{2} ,...,x_{n} \}$ be a given set of subjects; then a hesitant fuzzy set (HFS) on $X$ can be expressed as $H = \left\{ { < x,h_{Y(x)} > |x \in X} \right\}$, where $h_{Y(x)}$ is a nonempty finite subset on [0,1], representing several membership degrees belonging to set $H$.

In Definition 1, $h_{Y(x)}$ is an element of hesitant fuzzy set $H$, which is called a hesitant fuzzy number (HFN) and is expressed as $h = \{ \tau_{1} ,\tau_{2} ,...,\tau_{{l_{h} }} \} ,\tau \in [0,1],\lambda = 1,2,...,l_{h}$. This paper studies the extended form $h^{\prime}$ of HFN $h$, which is expressed as $h^{\prime} = \{ \tau^{\prime}_{1} ,\tau^{\prime}_{2} ,...,\tau^{\prime}_{{l^{\prime}_{h} }} \}$, where $\tau^{\prime}$ represents the score, $\tau^{\prime} \in [0,100],\lambda^{\prime} = 1,2,...,l^{\prime}_{h}$.

#### Definition 2 (Ref.^26^)

Let $h = \{ \tau_{i} |_{i = 1}^{{l_{h} }} \}$ be a HFN, and the number of elements in the HFN is represented by $l_{h}$; then its score function $s(h)$ is expressed as:

1 $$s(h) = \frac{1}{{l_{h} }}\sum\limits_{i = 1}^{{l_{h} }} {\tau_{i} } .$$

### Person-position matching and stable person-position matching

In person-position matching, let the position set be $A = \{ A_{1} ,A_{2} ,A_{3} ,...,A_{f} \}$ and the candidate set be $B = \{ B_{1} ,B_{2} ,B_{3} ,...,B_{g} \}$, where $A_{i}$ is the $i$-th position in $A$ and $B_{j}$ is the $j$-th candidate in $B$, $i \in F{ = }\{ 1,2,3,...,f\}$, $j \in G{ = }\{ 1,2,3,...,g\}$, $f \le g$.

#### Definition 3 (Ref.^27^)

For a mapping $\partial$:$A \cup B \to A \cup B$, if it satisfies: (1) $\partial (A_{i} ) \in B_{j}$; (2)$\partial (B_{j} ) \in A \cup \{ B_{j} \}$; (3) $\partial (A_{i} ) = B_{j}$ if and only if $\partial (B_{j} ) = A_{i}$, then $\partial$ is called a person-position matching, where $\partial (A_{i} ) = B_{j}$ means that $A_{i}$ matches with $B_{j}$ and $\partial (B_{j} ) = B_{j}$ means that $B_{j}$ does not match in $\partial$.

#### Definition 4 (Ref.^28^)

For a mapping $\partial$:$A \cup B \to A \cup B$, $\alpha_{ij}$ is the satisfaction of the manager of position $A_{i}$ to candidate $B_{j}$ and $\beta_{ij}$ is the satisfaction of candidate $B_{j}$ to position $A_{i}$. If there are not the following situations:

1. $\exists A_{i} ,A_{{i^{\prime}}} \in A$, $B_{j} ,B_{{j^{\prime}}} \in B$, such that $\partial (A_{i} ) = B_{{j^{\prime}}}$,$\partial (B_{j} ) = A_{{i^{\prime}}}$, and $\alpha_{{ij^{\prime}}} < \alpha_{ij}$, $\beta_{{i^{\prime}j}} < \beta_{ij}$;
2. $\exists B_{j} ,B_{{j^{\prime}}} \in B$, such that $\partial (A_{i} ) = B_{{j^{\prime}}}$, $\partial (B_{j} ) = B_{j}$, and $\alpha_{{ij^{\prime}}} < \alpha_{ij}$;
3. $\exists A_{i} ,A_{{i^{\prime}}} \in A$, such that $\partial (A_{i} ) = A_{i}$, $\partial (B_{j} ) = A_{{i^{\prime}}}$, and $\beta_{{i^{\prime}j}} < \beta_{ij}$;

then $\partial$ is called a stable person-position matching.

## Dynamic person-position matching decision under a hesitant fuzzy environment

### Problem description

Aiming at the dynamic person-position matching problem under a hesitant fuzzy environment, the position set is $A = \{ A_{1} ,A_{2} ,A_{3} ,...,A_{f} \}$ and the candidate set is $B = \{ B_{1} ,B_{2} ,B_{3} ,...,B_{f} \}$; the time period set considered is $T= \{t_{1} ,t_{2} ,...t_{q} \}$, where $t_{k}$ is the $k$-th time period, $k \in Q = \{ 1,2,...,q\}$. Assume that $A(t_{k} ){ = [}a_{ij} (t_{k} ){]}_{f \times g}$ is the hesitant fuzzy evaluation matrix of side $A$ to side $B$ at time $t_{k}$, $a_{ij} (t_{k} ) = \{ a_{ij}^{1} (t_{k} ),...,a_{ij}^{{\lambda_{a} }} (t_{k} ),...,a_{ij}^{{l_{{a_{ij} }} }} (t_{k} )\}$, where $a_{ij}^{{\lambda_{a} }} (t_{k} )$ represents the $\lambda_{a}$-th hesitant fuzzy evaluation value of the manager of position $A_{i}$ to candidate $B_{j}$ at time $t_{k}$, $a_{ij}^{{\lambda_{a} }} (t_{k} ) \in [0,100]$, $\lambda_{a} \in \{1,2,...,l_{{a_{ij} }} \}$; $B(t_{k} ){ = [}b_{ij} (t_{k} ){]}_{f \times g}$ is the hesitant fuzzy evaluation matrix of side $B$ to side $A$ at time $t_{k}$, $b_{ij} (t_{k} ) = \{ b_{ij}^{1} (t_{k} ),...,b_{ij}^{{\lambda_{b} }} (t_{k} ),...,b_{ij}^{{l_{{b_{ij} }} }} (t_{k} )\}$, where $b_{ij}^{{\lambda_{b} }} (t_{k} )$ represents the $\lambda_{b}$-th hesitant fuzzy evaluation value of candidate $B_{j}$ to position $A_{i}$ at time $t_{k}$,$b_{ij}^{{\lambda_{b} }} (t_{k} ) \in [0,100]$, $\lambda_{b} \in \{1,2,...,l_{{b_{ij} }} \}$.

**Figure 1:**
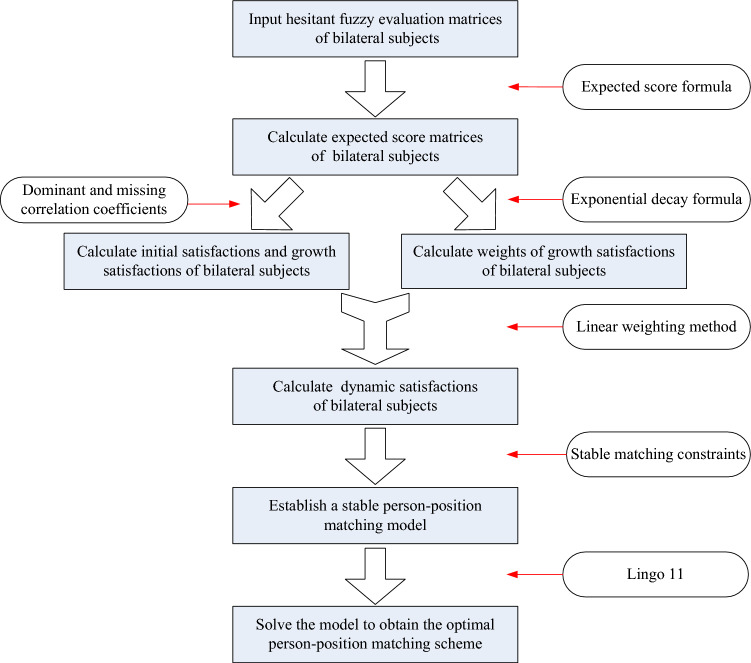
Dynamic person-position matching decision under a hesitant fuzzy environment.

The problems studied in this paper is to obtain the optimal dynamic person-position matching scheme according to hesitant fuzzy evaluation matrices. The solution idea is as follows: First, expected score matrices of bilateral subjects are calculated according to hesitant fuzzy evaluation matrices of positions and candidates. Then dynamic satisfactions of bilateral subjects are calculated by two correlation coefficients and exponential decay formula. On this basis, a stable person-position matching model is established and solved to obtain the optimal person-position matching scheme. The flow chart of dynamic person-position matching decision under a hesitant fuzzy environment is shown in Fig. 1.

### Calculation of dynamic satisfactions of candidates and positions

According to hesitant fuzzy evaluation matrices $A(t_{k} ){ = [}a_{ij} (t_{k} ){]}_{f \times g}$ and $B(t_{k} ){ = [}b_{ij} (t_{k} ){]}_{f \times g}$, extended expected scores $s_{{a_{ij} (t_{k} )}}$ and $s_{{b_{ij} (t_{k} )}}$ are obtained by Eq. (1) as follows:

2 $$s_{{a_{ij} (t_{k} )}} = \frac{1}{{l_{{a_{ij} }} }}\sum\limits_{z = 1}^{{l_{{a_{ij} }} }} {a_{ij}^{z} } (t_{k} ),$$

3 $$s_{{b_{ij} (t_{k} )}} = \frac{1}{{l_{{b_{ij} }} }}\sum\limits_{{z^{\prime} = 1}}^{{l_{{b_{ij} }} }} {b_{ij}^{{z^{\prime}}} } (t_{k} ),$$

By Eqs. (2) and (3), extended expected score matrices $S_{{a_{ij} (t_{k} )}} = {[}s_{{a_{ij} (t_{k} )}} {]}_{f \times g}$ and $S_{{b_{ij} (t_{k} )}} = {[}s_{{b_{ij} (t_{k} )}} {]}_{f \times g}$ are established.

In the following, the generalized priority method^29^ is used to calculate dynamic satisfactions of people and positions by introducing step length, generalized series and two preference relations. First, the definition of step length based on expected scores $s_{{a_{ij} (t_{k} )}}$ and $s_{{b_{ij} (t_{k} )}}$ is given.

#### Definition 5 (Ref.^28^)

Let the expected score $s_{{a_{ij} (t_{k} )}}$ of the manager of position $A_{i}$ to candidate $B_{j}$ at time $t_{k}$ be divided into $x + 1$ levels, that is, the evaluation value of the manager of position $A_{i}$ at time $t_{k}$ is at most superior to or inferior to that of candidate $B_{j}$ $x$ levels; then the step length of the manager of position $A_{i}$ to candidate $B_{j}$ at time $t_{k}$ is expressed by $\gamma_{ij}^{A} (t_{k} )$, which is calculated as follows:

4 $$\gamma_{ij}^{A} (t_{k} ) = \frac{{\mathop {\max }\limits_{1 \le i \le f,1 \le j \le g} s_{{a_{ij} (t_{k} )}} - \mathop {\min }\limits_{1 \le i \le f,1 \le j \le g} s_{{a_{ij} (t_{k} )}} }}{x},$$

Then the expected score $s_{{b_{ij} (t_{k} )}}$ of candidate $B_{j}$ to position $A_{i}$ at time $t_{k}$ is also divided into $x + 1$ levels, and the step length of candidate $B_{j}$ to position $A_{i}$ at time $t_{k}$ is expressed by $\gamma_{ij}^{B} (t_{k} )$, which is calculated as follows:

5 $$\gamma_{ij}^{B} (t_{k} ) = \frac{{\mathop {\max }\limits_{1 \le i \le f,1 \le j \le g} s_{{b_{ij} (t_{k} )}} - \mathop {\min }\limits_{1 \le i \le f,1 \le j \le g} s_{{b_{ij} (t_{k} )}} }}{x}.$$

Then, the definition of generalized series is introduced based on the step length.

#### Definition 6 (Ref.^28^)

Assume that expected scores of candidates $B_{j}$ and $B_{{j^{\prime}}}$ with respect to position $A_{i}$ at time $t_{k}$ are $s_{{a_{ij} (t_{k} )}}$ and $s_{{a_{{ij^{\prime}}} (t_{k} )}}$; then the level of two candidates $B_{j}$ and $B_{{j^{\prime}}}$ obtained by comparison is called the generalized series, expressed as $r_{{ijj^{\prime}}} (t_{k} )$, which is calculated as follows:

6 $$r_{{ijj^{\prime}}} (t_{k} ) = \frac{{s_{{a_{ij} (t_{k} )}} - s_{{a_{{ij^{\prime}}} (t_{k} )}} }}{{\gamma_{ij}^{A} (t_{k} )}},$$

Assuming that expected scores of managers of positions $A_{i}$ and $A_{{i^{\prime}}}$ with respect to candidate $B_{j}$ at time $t_{k}$ are $s_{{b_{ij} (t_{k} )}}$ and $s_{{b_{{i^{\prime}j}} (t_{k} )}}$, respectively, $r_{{ii^{\prime}j}} (t_{k} )$ is calculated as follows:

7 $$r_{{ii^{\prime}j}} (t_{k} ) = \frac{{s_{{b_{ij} (t_{k} )}} - s_{{b_{{i^{\prime}j}} (t_{k} )}} }}{{\gamma_{ij}^{B} (t_{k} )}}.$$

In Eq. (6), $r_{{ijj^{\prime}}} (t_{k} )$ indicates the rank of expected scores given by candidates $B_{j}$ and $B_{{j^{\prime}}}$ with respect to position $A_{i}$ at time $t_{k}$; $s_{{a_{ij} (t_{k} )}} \mathop \succ \limits^{{r_{{ijj^{\prime}}} (t_{k} )}} s_{{a_{{ij^{\prime}}} (t_{k} )}}$ is understood that the expected score of candidate $B_{j}$ is $r_{{ijj^{\prime}}} (t_{k} )$ levels better than that of candidate $B_{{j^{\prime}}}$ with respect to position $A_{i}$ at time $t_{k}$; $s_{{a_{ij} (t_{k} )}} \mathop \prec \limits^{{r_{{ijj^{\prime}}} (t_{k} )}} s_{{a_{{ij^{\prime}}} (t_{k} )}}$ is understood that the expected score of candidate $B_{j}$ is $r_{{ijj^{\prime}}} (t_{k} )$ levels worse than that of candidate $B_{{j^{\prime}}}$ with respect to position $A_{i}$ at time $t_{k}$; $s_{{a_{ij} (t_{k} )}} \approx s_{{a_{{ij^{\prime}}} (t_{k} )}}$ is understood that the expected score of candidate $B_{j}$ is equal to that of candidate $B_{{j^{\prime}}}$ with respect to position $A_{i}$ at time $t_{k}$. $s_{{a_{ij} (t_{k} )}} ?s_{{a_{{ij^{\prime}}} (t_{k} )}}$ is understood that the level of the expected score of candidates $B_{j}$ and $B_{{j^{\prime}}}$ with respect to position $A_{i}$ at time $t_{k}$ is missing. The meaning of $r_{{ii^{\prime}j}} (t_{k} )$ in Eq. (7) is similar to that of $r_{{ijj^{\prime}}} (t_{k} )$ in Eq. (6).

Next, the generalized priority method is used to transform expected scores into satisfaction means of managers of positions and candidates.

#### Definition 7 (Ref.^30^)

Assume that expected scores are divided into $x + 1$ levels; $\{ \succ , \prec , \approx ,?\}$ denotes the set of preference relations, which can be understood as {better, worse, same, missing}, $S,S^{\prime} \in \{ \succ , \prec , \approx ,?\}$, and $d(S,S^{\prime})$ represents the distance between score preference relations $S$ and $S^{\prime}$. Then

$$\Delta_{{\mathop \succ \limits^{x} }} \max = \max \{ d(S,\mathop \succ \limits^{x} )\left| {S \in } \right.\{ \mathop \succ \limits^{1} ,\mathop \succ \limits^{2} ,...\mathop \succ \limits^{x} ,\mathop \prec \limits^{1} ,\mathop \prec \limits^{2} ,...,\mathop \prec \limits^{x} , \approx ,?\} \} ,$$

$$\Delta_{{\mathop \succ \limits^{x} }} \min = \min \{ d(S,\mathop \succ \limits^{x} )\left| {S \in } \right.\{ \mathop \succ \limits^{1} ,\mathop \succ \limits^{2} ,...\mathop \succ \limits^{x} ,\mathop \prec \limits^{1} ,\mathop \prec \limits^{2} ,...,\mathop \prec \limits^{x} , \approx ,?\} \} .$$

wherein, $\Delta_{{\mathop \succ \limits^{x} }} \max$ denotes the maximum among distances between preference relations $S$ and $\mathop \succ \limits^{x}$ of the dominant score, and $\Delta_{{\mathop \succ \limits^{x} }} \min$ denotes the minimum among distances between preference relations $S$ and $\mathop \succ \limits^{x}$ of the dominant score.

#### *Remark 1*

According to literature^29^, distances among expected score preference relations are shown in Table 2, where $a$ is the independent variable, satisfying $a > 0$.

**Table 2:** Distances between expected score preference relations.

	$\succ$	$\prec$	$?$	$\approx$
$\succ$	$0$	$2a$	$\frac{5a}{3}$	$a$
$\prec$	$2a$	$0$	$\frac{5a}{3}$	$a$
$?$	$\frac{5a}{3}$	$\frac{5a}{3}$	$0$	$\frac{4a}{3}$
$\approx$	$a$	$a$	$\frac{4a}{3}$	$0$

According to literature^29^, the dominant correlation coefficient of the score preference relation $\eta_{{\mathop \succ \limits^{x} }} (S^{\prime})$ is calculated as follows:

8 $$\eta_{{\mathop \succ \limits^{x} }} (S^{\prime}) = \frac{{\Delta_{{\mathop \succ \limits^{x} }} \min + \delta \Delta_{{\mathop \succ \limits^{x} }} \max }}{{d(S,\mathop \succ \limits^{x} ) + \delta \Delta_{{\mathop \succ \limits^{x} }} \max }},$$

where $\delta \in [0,1]$ is the resolution coefficient, and usually satisfies $\delta = 0.5$.

Based on Definition 7, the definition of the missing score preference relation is as follows:

#### **Definition 8**

Assume that expected scores are divided into $x + 1$ levels, score preference relations are $S,S^{\prime} \in \{ \succ , \prec , \approx ,?\}$, and $d(S,S^{\prime})$ represents the distance between score preference relations $S$ and $S^{\prime}$. Then

$$\Delta_{?} \max = \max \{ d(S,?)\left| {S \in } \right.\{ \mathop \succ \limits^{1} ,\mathop \succ \limits^{2} ,...\mathop \succ \limits^{x} ,\mathop \prec \limits^{1} ,\mathop \prec \limits^{2} ,...,\mathop \prec \limits^{x} , \approx ,?\} \} ,$$

$$\Delta_{?} \min = \min \{ d(S,?)\left| {S \in } \right.\{ \mathop \succ \limits^{1} ,\mathop \succ \limits^{2} ,...\mathop \succ \limits^{x} ,\mathop \prec \limits^{1} ,\mathop \prec \limits^{2} ,...,\mathop \prec \limits^{x} , \approx ,?\} \} .$$

wherein, $\Delta_{?} \max$ denotes the maximum among distances between preference relations $S$ and $?$ of the missing score and $\Delta_{?} \min$ denotes the minimum among distances between preference relations $S$ and $?$ of the missing score.

On this basis, the missing correlation coefficient of the score preference relation $\eta_{?} (S^{\prime})$ is calculated as follows:

9 $$\eta_{?} (S^{\prime}) = \frac{{\Delta_{?} \min + \delta \Delta_{?} \max }}{{d(S,?) + \delta \Delta_{?} \max }},$$

where $\delta \in [0,1]$ is the resolution coefficient, and usually satisfies $\delta = 0.5$.

By Eq. (8) and Table 2, the dominant correlation coefficient is calculated as follows:

10 $$\left\{ \begin{aligned} & \eta_{{\mathop \succ \limits^{x} }} (\mathop \succ \limits^{\lambda } ) = \frac{x}{2x - \lambda } , \hfill \\ & \eta_{{\mathop \succ \limits^{x} }} (\mathop \prec \limits^{\lambda } ) = \frac{x}{2x + \lambda }, \hfill \\ & \eta_{{\mathop \succ \limits^{x} }} (?) = 0.375, \hfill \\ & \eta_{{\mathop \succ \limits^{x} }} ( \approx ) = 0.5, \hfill \\ \end{aligned} \right.$$

The generalized equal series $r_{{ijj^{\prime}}} (t_{k} )$ is substituted into Eq. (10), and the relative satisfaction $\alpha_{{_{{ijj^{\prime}}} }}^{ \succ } (t_{k} )$ of the manager of position $A_{i}$ to candidate $B_{j}$ based on the dominant correlation coefficient at time $t_{k}$ is calculated as follows:

11 $$\alpha _{{_{{ijj^{\prime}}} }}^{ \succ } (t_{k} ) = \left\{ {\begin{array}{lll} {\frac{x}{{2x - r_{{ijj^{\prime}}} (t_{k} )}}{\kern 1pt} {\kern 1pt} {\kern 1pt} {\kern 1pt} ,} & \quad {s_{{a_{{ij}} (t_{k} )}} \mathop \succ \limits^{{r_{{ijj^{\prime}}} (t_{k} )}} s_{{a_{{ij^{\prime}}} (t_{k} )}} ,} & \quad {j \ne j^{\prime},} \\ {0.5,} & \quad{s_{{a_{{ij}} (t_{k} )}} \approx s_{{a_{{ij^{\prime}}} (t_{k} )}} ,} & \quad {j \ne j^{\prime},} \\ {{\kern 1pt} 0.375{\kern 1pt} {\kern 1pt} ,} & \quad{s_{{a_{{ij}} (t_{k} )}} ?s_{{a_{{ij^{\prime}}} (t_{k} )}} ,} & \quad{j \ne j^{\prime},} \\ {\frac{x}{{2x + {\kern 1pt} r_{{ijj^{\prime}}} (t_{k} )}},} & \quad{s_{{a_{{ij}} (t_{k} )}} \mathop \prec \limits^{{r_{{ijj^{\prime}}} (t_{k} )}} s_{{a_{{ij^{\prime}}} (t_{k} )}} ,} &\quad {j \ne j^{\prime}.} \\ \end{array} } \right.$$

Similarly, by Eq. (9) and Table 2, the missing correlation coefficient is calculated as follows:

12 $$\left\{ \begin{aligned} & \eta_{?} (\mathop \succ \limits^{\lambda } ) = \frac{5x}{{13x - 8\lambda }},{\kern 1pt} \hfill \\ & {\kern 1pt} {\kern 1pt} \eta_{?} (\mathop \prec \limits^{\lambda } ) = \frac{5x}{{13x + 8\lambda }}{\kern 1pt} , \hfill \\ & \eta_{?} (?) = 1,{\kern 1pt} \hfill \\ & \eta_{?} ( \approx ) = \frac{5}{13}, \hfill \\ \end{aligned} \right.$$

The generalized equal series $r_{{ijj^{\prime}}} (t_{k} )$ is substituted into Eq. (12), and the relative satisfaction $\alpha_{{_{{ijj^{\prime}}} }}^{?} (t_{k} )$ of the manager of position $A_{i}$ to candidate $B_{j}$ based on the missing correlation coefficient at time $t_{k}$ is calculated as follows:

13 $$\alpha _{{_{{ijj^{\prime}}} }}^{?} (t_{k} ) = \left\{ {\begin{array}{lll} {\frac{{5x}}{{13x - 8r_{{ijj^{\prime}}} (t_{k} )}}{\kern 1pt} ,} & \quad {s_{{a_{{ij}} (t_{k} )}} \mathop \succ \limits^{{r_{{ijj^{\prime}}} (t_{k} )}} s_{{a_{{ij^{\prime}}} (t_{k} )}} ,} & \quad {j \ne j^{\prime},} \\ {{\kern 1pt} {\kern 1pt} {\kern 1pt} {\kern 1pt} {\kern 1pt} \frac{5}{{13}},} & \quad {s_{{a_{{ij}} (t_{k} )}} \approx s_{{a_{{ij^{\prime}}} (t_{k} )}} ,} &\quad {j \ne j^{\prime},} \\ {0.375{\kern 1pt} ,} & \quad {s_{{a_{{ij}} (t_{k} )}} ?s_{{a_{{ij^{\prime}}} (t_{k} )}} ,} &\quad {j \ne j^{\prime},} \\ {\frac{{5x}}{{13x{\text{ + }}{\kern 1pt} 8r_{{ijj^{\prime}}} (t_{k} )}}{\kern 1pt} ,} & \quad {s_{{a_{{ij}} (t_{k} )}} \mathop \prec \limits^{{r_{{ijj^{\prime}}} (t_{k} )}} s_{{a_{{ij^{\prime}}} (t_{k} )}} ,} & \quad {j \ne j^{\prime}.} \\ \end{array} } \right.$$

Furthermore, the correlation parameter $\theta$($0 \le \theta \le 1$) is introduced to calculate the satisfaction mean of the manager of position $A_{i}$ to candidate $B_{j}$ at time $t_{k}$, i.e.,

14 $$\alpha_{ij} \left( {t_{k} } \right){ = }\frac{\theta }{g - 1}\sum\limits_{{j \in g,j \ne j^{\prime}}} {\alpha_{{ijj^{\prime}}}^{ \succ } } \left( {t_{k} } \right) + \frac{1 - \theta }{{g - 1}}\sum\limits_{{j \in g,j \ne j^{\prime}}} {\alpha_{{ijj^{\prime}}}^{?} } \left( {t_{k} } \right)$$

By Eq. (14), the satisfaction mean matrix $\Phi (t_{k} ) = [\alpha_{ij} \left( {t_{k} } \right)]_{f \times g}$ of side $A$ to side $B$ at time $t_{k}$ is obtained.

Then, considering the change of growth satisfaction means, the growth satisfaction mean matrix $\Delta \Phi (t_{k} )$ of side $A$ to side $B$ at time $t_{k}$ is calculated according to satisfaction mean matrix $\Phi (t_{k} )$ as follows:

15 $$\Delta \Phi (t_{k} ) = [\Delta \alpha_{ij} \left( {t_{k} } \right)]_{f \times g} = \Phi (t_{k + 1} ) - \Phi (t_{k} ) = [\alpha_{ij} \left( {t_{k + 1} } \right) - \alpha_{ij} \left( {t_{k} } \right)]_{f \times g} .$$

Similarly, the generalized equal series $r_{{ii^{\prime}j}} (t_{k} )$ is substituted into Eq. (10), and the relative satisfaction $\beta_{{_{{ii^{\prime}j}} }}^{ \succ } (t_{k} )$ of candidate $B_{j}$ to position $A_{i}$ based on the dominant correlation coefficient at time $t_{k}$ is calculated as follows:

16 $$\beta _{{_{{ii^{\prime}j}} }}^{ \succ } (t_{k} ) = \left\{ {\begin{array}{lll} {\frac{x}{{2x - r_{{ii^{\prime}j}} (t_{k} )}},} & \quad {s_{{b_{{ij}} (t_{k} )}} \mathop \succ \limits^{{r_{{ii^{\prime}j}} (t_{k} )}} s_{{b_{{i^{\prime}j}} (t_{k} )}} ,} & \quad{i \ne i^{\prime},} \\ {{\kern 1pt} {\kern 1pt} 0.5{\kern 1pt} ,} &\quad {s_{{b_{{ij}} (t_{k} )}} \approx s_{{b_{{i^{\prime}j}} (t_{k} )}} ,} & \quad{j \ne j^{\prime},} \\ {0.375,} & \quad {s_{{b_{{ij}} (t_{k} )}} ?s_{{b_{{i^{\prime}j}} (t_{k} )}} ,} & \quad{j \ne j^{\prime},} \\ {\frac{x}{{2x{\text{ + }}{\kern 1pt} r_{{ii^{\prime}j}} (t_{k} )}},} &\quad {s_{{b_{{ij}} (t_{k} )}} \mathop \prec \limits^{{r_{{ifj}} (t_{k} )}} s_{{b_{{i^{\prime}j}} (t_{k} )}} ,} & \quad {i \ne i^{\prime},} \\ \end{array} } \right.$$

The generalized equal series $r_{{ii^{\prime}j}} (t_{k} )$ is substituted into Eq. (12), and the relative satisfaction $\beta_{{_{{ii^{\prime}j}} }}^{?} (t_{k} )$ of candidate $B_{j}$ to position $A_{i}$ based on the missing correlation coefficient at time $t_{k}$ is calculated as follows:

17 $$\beta _{{_{{ii^{\prime}j}} }}^{?} (t_{k} ) = \left\{ {\begin{array}{lll} {\frac{{5x}}{{13x - 8r_{{ii^{\prime}j}} (t_{k} )}},} & \quad {s_{{b_{{ij}} (t_{k} )}} \mathop \succ \limits^{{r_{{ii^{\prime}j}} (t_{k} )}} s_{{b_{{i^{\prime}j}} (t_{k} )}} ,} & \quad {i \ne i^{\prime},} \\ {\frac{5}{{13}}{\kern 1pt} {\kern 1pt} ,{\kern 1pt} } & \quad {s_{{b_{{ij}} (t_{k} )}} \approx s_{{b_{{i^{\prime}j}} (t_{k} )}} ,} &\quad {i \ne i^{\prime},} \\ {{\kern 1pt} 0.375,{\kern 1pt} } & \quad {s_{{b_{{ij}} (t_{k} )}} ?s_{{b_{{i^{\prime}j}} (t_{k} )}} ,} & \quad{j \ne j^{\prime},} \\ \hline {\frac{{5x}}{{13x{\text{ + }}{\kern 1pt} 8r_{{ii^{\prime}j}} (t_{k} )}}{\kern 1pt} {\kern 1pt} ,} &\quad {s_{{b_{{ij}} (t_{k} )}} \mathop \prec \limits^{{r_{{ii^{\prime}j}} (t_{k} )}} s_{{b_{{i^{\prime}j}} (t_{k} )}} ,} &\quad {i \ne i^{\prime}.} \\ \end{array} } \right.$$

Furthermore, the correlation parameter $\theta$($0 \le \theta \le 1$) is introduced to calculate the satisfaction mean of candidate $B_{j}$ to position $A_{i}$ at time $t_{k}$, i.e.,

18 $$\beta_{ij} \left( {t_{k} } \right){ = }\frac{\theta }{f - 1}\sum\limits_{{i \in f,i \ne i^{\prime}}} {\beta_{{_{{ii^{\prime}j}} }}^{ \succ } } \left( {t_{k} } \right) + \frac{1 - \theta }{{f - 1}}\sum\limits_{{i \in f,i \ne i^{\prime}}} {\beta_{{_{{ii^{\prime}j}} }}^{?} } \left( {t_{k} } \right),$$

By Eq. (18), the satisfaction mean matrix $\Psi (t_{k} ) = [\beta_{ij} \left( {t_{k} } \right)]_{f \times g}$ of side $B$ to side $A$ at time $t_{k}$ is obtained.

Then, considering the change of growth satisfaction means, the growth satisfaction mean matrix $\Delta \Psi (t_{k} ) = [\Delta \beta_{ij} \left( {t_{k} } \right)]_{f \times g}$ of side $B$ to side $A$ at time $t_{k}$ is calculated as follows:

19 $$\Delta \Psi (t_{k} ) = [\Delta \beta_{ij} (t_{k} )]_{f \times g} = \Psi (t_{k + 1} ) - \Psi (t_{k} ) = [\beta_{ij} \left( {t_{k + 1} } \right) - \beta_{ij} (t_{k} )]_{f \times g} .$$

According to literature^30^, the exponential decay formula is used to determine growth satisfaction weights of bilateral subjects at time $t_{k}$, i.e.,

20 $$\omega_{{t_{k} }}^{\Delta } = \frac{{e^{{\rho t_{k} }} }}{{\sum\nolimits_{{t_{{k^{\prime}}} = 2}}^{q} {e^{{\rho t_{{k^{\prime}}} }} } }},t_{k} = 2,3,...,q,$$

where, $\rho$($0 \le \rho \le 1$) is the attenuation coefficient, which reflects the change of the importance of growth satisfactions over time.

Finally, according to the initial satisfaction matrix $\Phi (t_{1} )$, the growth satisfaction matrix $\Delta \Phi (t_{k} )$ and weights $\omega_{2}^{\Delta } ,...,\omega_{{t_{k} }}^{\Delta } ,...,\omega_{q}^{\Delta }$, the dynamic satisfaction matrix $\mathop{\Phi }\limits^{\leftrightarrow} = [\mathop{\alpha }\limits^{\leftrightarrow} _{ij} ]_{f \times g}$ of side $A$ to side $B$ is obtained as follows:

21 $$\begin{aligned} \mathop{\Phi }\limits^{\leftrightarrow} & = \Phi (t_{1} ) + \omega_{2}^{\Delta } \Delta \Phi_{1} + \cdots + \omega_{{t_{k} }}^{\Delta } \Delta \Phi_{k - 1} + \cdots + \omega_{q}^{\Delta } \Delta \Phi_{q - 1} \hfill \\ & = [\alpha_{ij} \left( {t_{1} } \right)]_{f \times g} + \omega_{2}^{\Delta } [\alpha_{ij} \left( {t_{2} } \right) - \alpha_{ij} \left( {t_{1} } \right)]_{f \times g} + \cdots + \omega_{{t_{k} }}^{\Delta } [\alpha_{ij} \left( {t_{k} } \right) - \alpha_{ij} \left( {t_{k - 1} } \right)]_{f \times g} {\kern 1pt} \\ & \quad + \cdots + \omega_{q}^{\Delta } [\alpha_{ij} \left( {t_{q} } \right) - \alpha_{ij} \left( {t_{q - 1} } \right)]_{f \times g} \hfill \\ \end{aligned}$$

where $$0 \le \omega_{2}^{\Delta } ,...,\omega_{{t_{k} }}^{\Delta } ,...,\omega_{q}^{\Delta } \le 1$$, $$\sum\nolimits_{{t_{k} = 2}}^{q} {\omega_{{t_{k} }}^{\Delta } } = 1$$.

According to the initial satisfaction matrix $\Psi (t_{1} )$, the growth satisfaction matrix $\Delta \Psi (t_{k} )$ and weights $\omega_{2}^{\Delta } ,...,\omega_{{t_{k} }}^{\Delta } ,...,\omega_{q}^{\Delta }$, the dynamic satisfaction matrix $\mathop{\Psi }\limits^{\leftrightarrow} = [\mathop{\beta }\limits^{\leftrightarrow} _{ij} ]_{f \times g}$ of side $B$ to side $A$ is obtained as follows:

22 $$\begin{aligned} \mathop{\Psi }\limits^{\leftrightarrow} & = \Psi (t_{1} ) + \omega_{2}^{\Delta } \Delta \Psi_{1} + \cdots + \omega_{{t_{k} }}^{\Delta } \Delta \Psi_{k - 1} + \cdots + \omega_{q}^{\Delta } \Delta \Psi_{q - 1} \hfill \\ & = [\beta_{ij} \left( {t_{1} } \right)]_{f \times g} + \omega_{2}^{\Delta } [\beta_{ij} \left( {t_{2} } \right) - \beta_{ij} \left( {t_{1} } \right)]_{f \times g} + \cdots + \omega_{{t_{k} }}^{\Delta } [\beta_{ij} \left( {t_{k} } \right) - \beta_{ij} \left( {t_{k - 1} } \right)]_{f \times g} {\kern 1pt} {\kern 1pt} \\ & \quad + \cdots + \omega_{q}^{\Delta } [\beta_{ij} \left( {t_{q} } \right) - \beta_{ij} \left( {t_{q - 1} } \right)]_{f \times g} \hfill \\ \end{aligned}$$

### Establishment of stable person-position matching model considering dynamic satisfactions

First, assume that the stable person-position matching matrix is $X = [x_{ij} ]_{f \times g}$, where $$x_{ij} = \left\{ \begin{gathered} 1,\eta (a_{i} ) = b_{j} \hfill \\ 0,\eta (a_{i} ) \ne b_{j} \hfill \\ \end{gathered} \right.$$. Then, according to dynamic satisfaction matrices $\mathop{\Phi }\limits^{\leftrightarrow} = [\mathop{\alpha }\limits^{\leftrightarrow} _{ij} ]_{f \times g}$ and $\mathop{\Psi }\limits^{\leftrightarrow} = [\mathop{\beta }\limits^{\leftrightarrow} _{ij} ]_{f \times g}$ and matching matrix $X = [x_{ij} ]_{f \times g}$, the following multi-objective model (M-1) is established with goals of maximizing dynamic satisfactions of managers of positions and candidates and constraints of stable matching:

$$({\text{M}} - 1)\left\{ \begin{aligned} & {\text{Max}}{\kern 1pt} {\kern 1pt} {\kern 1pt} {\kern 1pt} {\kern 1pt} {\kern 1pt} D_{A} = \sum\limits_{{i = 1}}^{f} {\sum\limits_{{j = 1}}^{g} {\overset{\lower0.5em\hbox{$\smash{\scriptscriptstyle\leftrightarrow}$}} {\alpha } _{{ij}} x_{{ij}} } } , \hfill \\ & {\text{Max}}{\kern 1pt} {\kern 1pt} {\kern 1pt} {\kern 1pt} {\kern 1pt} {\kern 1pt} D_{B} = \sum\limits_{{i = 1}}^{f} {\sum\limits_{{j = 1}}^{g} {\overset{\lower0.5em\hbox{$\smash{\scriptscriptstyle\leftrightarrow}$}} {\beta } _{{ij}} x_{{ij}} } } , \hfill \\ & {\text{s.t.}} {\kern 1pt} {\kern 1pt} {\kern 1pt} {\kern 1pt} {\kern 1pt} {\kern 1pt} {\kern 1pt} {\kern 1pt} {\kern 1pt} {\kern 1pt} {\kern 1pt} {\kern 1pt} {\kern 1pt} {\kern 1pt} {\kern 1pt} {\kern 1pt} {\kern 1pt} \sum\limits_{{j = 1}}^{g} {x_{{ij}} \le 1,i \in F} , \hfill \\ & {\kern 1pt} {\kern 1pt} {\kern 1pt} {\kern 1pt} {\kern 1pt} {\kern 1pt} {\kern 1pt} {\kern 1pt} {\kern 1pt} {\kern 1pt} {\kern 1pt} {\kern 1pt} {\kern 1pt} {\kern 1pt} {\kern 1pt} {\kern 1pt} {\kern 1pt} {\kern 1pt} {\kern 1pt} {\kern 1pt} {\kern 1pt} {\kern 1pt} {\kern 1pt} {\kern 1pt} {\kern 1pt} {\kern 1pt} {\kern 1pt} {\kern 1pt} {\kern 1pt} {\kern 1pt} {\kern 1pt} \sum\limits_{{i = 1}}^{f} {x_{{ij}} \le 1,j \in G} , \hfill \\ & {\kern 1pt} {\kern 1pt} {\kern 1pt} {\kern 1pt} {\kern 1pt} {\kern 1pt} {\kern 1pt} {\kern 1pt} {\kern 1pt} {\kern 1pt} {\kern 1pt} {\kern 1pt} {\kern 1pt} {\kern 1pt} {\kern 1pt} {\kern 1pt} {\kern 1pt} {\kern 1pt} {\kern 1pt} {\kern 1pt} {\kern 1pt} {\kern 1pt} {\kern 1pt} {\kern 1pt} {\kern 1pt} {\kern 1pt} {\kern 1pt} {\kern 1pt} {\kern 1pt} {\kern 1pt} {\kern 1pt} x_{{ij}} + \sum\limits_{{j^{\prime}:\overset{\lower0.5em\hbox{$\smash{\scriptscriptstyle\leftrightarrow}$}} {\alpha } _{{ij^{\prime}}} > \overset{\lower0.5em\hbox{$\smash{\scriptscriptstyle\leftrightarrow}$}} {\alpha } _{{ij}} }} {x_{{ij^{\prime}}} + \sum\limits_{{i^{\prime}:\overset{\lower0.5em\hbox{$\smash{\scriptscriptstyle\leftrightarrow}$}} {\beta } _{{i^{\prime}j}} > \overset{\lower0.5em\hbox{$\smash{\scriptscriptstyle\leftrightarrow}$}} {\beta } _{{ij}} }} {x_{{i^{\prime}j}} \ge 1,i \in F,j \in G} } , \hfill \\ & {\kern 1pt} {\kern 1pt} {\kern 1pt} {\kern 1pt} {\kern 1pt} {\kern 1pt} {\kern 1pt} {\kern 1pt} {\kern 1pt} {\kern 1pt} {\kern 1pt} {\kern 1pt} {\kern 1pt} {\kern 1pt} {\kern 1pt} {\kern 1pt} {\kern 1pt} {\kern 1pt} {\kern 1pt} {\kern 1pt} {\kern 1pt} {\kern 1pt} {\kern 1pt} {\kern 1pt} {\kern 1pt} {\kern 1pt} {\kern 1pt} {\kern 1pt} {\kern 1pt} {\kern 1pt} x_{{ij}} \in \{ 0,1\} ,i \in F,j \in G. \hfill \\ \end{aligned} \right.$$

wherein, ${\varvec{Max}}{\kern 1pt} {\kern 1pt} {\kern 1pt} {\kern 1pt} {\kern 1pt} {\kern 1pt} D_{A} = \sum\limits_{i = 1}^{f} {\sum\limits_{j = 1}^{g} {\mathop{\alpha }\limits^{\leftrightarrow} _{ij} x_{ij} } }$ denotes maximizing dynamic satisfactions of managers of positions, and ${\varvec{Max}}{\kern 1pt} {\kern 1pt} {\kern 1pt} {\kern 1pt} {\kern 1pt} {\kern 1pt} D_{B} = \sum\limits_{i = 1}^{f} {\sum\limits_{j = 1}^{g} {\mathop{\beta }\limits^{\leftrightarrow} _{ij} x_{ij} } }$ denotes maximizing dynamic satisfactions of candidates.

### Solution of stable person-position matching model considering dynamic satisfactions

For objective functions $D_{A}$ and $D_{B}$, considering that dimensions of $\mathop{\alpha }\limits^{\leftrightarrow} _{ij}$ and $\mathop{\beta }\limits^{\leftrightarrow} _{ij}$ are consistent, the multi-objective model (M-1) is transformed into a single-objective model (M-2) by the linear weighting method:

$${(\text{M}}{ - }{2)}\left\{ \begin{aligned} & {\text{Max}}{\kern 1pt} {\kern 1pt} {\kern 1pt} {\kern 1pt} {\kern 1pt} {\kern 1pt} D = \omega_{1} \sum\limits_{i = 1}^{f} {\sum\limits_{j = 1}^{g} {\mathop{\alpha }\limits^{\leftrightarrow} _{ij} x_{ij} + \omega_{2} \sum\limits_{i = 1}^{f} {\sum\limits_{j = 1}^{g} {\mathop{\beta }\limits^{\leftrightarrow} _{ij} x_{ij} } } } } , \hfill \\ & {\text{s.t.}} {\kern 1pt} {\kern 1pt} {\kern 1pt} {\kern 1pt} {\kern 1pt} {\kern 1pt} {\kern 1pt} {\kern 1pt} {\kern 1pt} {\kern 1pt} {\kern 1pt} {\kern 1pt} {\kern 1pt} {\kern 1pt} {\kern 1pt} {\kern 1pt} {\kern 1pt} {\kern 1pt} \sum\limits_{j = 1}^{g} {x_{ij} \le 1,i \in F} , \hfill \\ & {\kern 1pt} {\kern 1pt} {\kern 1pt} {\kern 1pt} {\kern 1pt} {\kern 1pt} {\kern 1pt} {\kern 1pt} {\kern 1pt} {\kern 1pt} {\kern 1pt} {\kern 1pt} {\kern 1pt} {\kern 1pt} {\kern 1pt} {\kern 1pt} {\kern 1pt} {\kern 1pt} {\kern 1pt} {\kern 1pt} {\kern 1pt} {\kern 1pt} {\kern 1pt} {\kern 1pt} {\kern 1pt} {\kern 1pt} {\kern 1pt} {\kern 1pt} {\kern 1pt} {\kern 1pt} {\kern 1pt} \sum\limits_{i = 1}^{f} {x_{ij} \le 1,j \in G} , \hfill \\ & {\kern 1pt} {\kern 1pt} {\kern 1pt} {\kern 1pt} {\kern 1pt} {\kern 1pt} {\kern 1pt} {\kern 1pt} {\kern 1pt} {\kern 1pt} {\kern 1pt} {\kern 1pt} {\kern 1pt} {\kern 1pt} {\kern 1pt} {\kern 1pt} {\kern 1pt} {\kern 1pt} {\kern 1pt} {\kern 1pt} {\kern 1pt} {\kern 1pt} {\kern 1pt} {\kern 1pt} {\kern 1pt} {\kern 1pt} {\kern 1pt} {\kern 1pt} {\kern 1pt} {\kern 1pt} {\kern 1pt} x_{ij} + \sum\limits_{{j^{\prime}:\mathop{\alpha }\limits^{\leftrightarrow} _{{ij^{\prime}}} > \mathop{\alpha }\limits^{\leftrightarrow} _{ij} }} {x_{{ij^{\prime}}} + \sum\limits_{{i^{\prime}:\mathop{\beta }\limits^{\leftrightarrow} _{{i^{\prime}j}} > \mathop{\beta }\limits^{\leftrightarrow} _{ij} }} {x_{{i^{\prime}j}} \ge 1,i \in F,j \in G} } ,{\kern 1pt} {\kern 1pt} {\kern 1pt} {\kern 1pt} {\kern 1pt} {\kern 1pt} {\kern 1pt} \hfill \\ &{\kern 1pt} {\kern 1pt} {\kern 1pt} {\kern 1pt} {\kern 1pt} {\kern 1pt} {\kern 1pt} {\kern 1pt} {\kern 1pt} {\kern 1pt} {\kern 1pt} {\kern 1pt} {\kern 1pt} {\kern 1pt} {\kern 1pt} {\kern 1pt} {\kern 1pt} {\kern 1pt} {\kern 1pt} {\kern 1pt} {\kern 1pt} {\kern 1pt} {\kern 1pt} {\kern 1pt} {\kern 1pt} {\kern 1pt} {\kern 1pt} {\kern 1pt} {\kern 1pt} {\kern 1pt} {\kern 1pt} x_{ij} \in \{ 0,1\} ,i \in F,j \in G. \hfill \\ \end{aligned} \right.$$

where, $\omega_{1}$ and $\omega_{2}$ are weights of objective functions $D_{A}$ and $D_{B}$, respectively, satisfying $0 \le \omega_{1} ,\omega_{2} \le 1$, $\omega_{1} + \omega_{2} = 1$.

### Decision steps for dynamic person-position matching under a hesitant fuzzy environment

For the dynamic person-position matching problem under a hesitant fuzzy environment, steps of the proposed decision method are as follows:

Step 1.: According to hesitant fuzzy evaluation matrices $A(t_{k} ){ = [}a_{ij} (t_{k} ){]}_{f \times g}$ and $B(t_{k} ){ = [}b_{ij} (t_{k} ){]}_{f \times g}$ at time $t_{k}$, expected score matrices $S_{{a_{ij} (t_{k} )}} { = [}s_{{a_{ij} (t_{k} )}} {]}_{f \times g}$ and $S_{{b_{ij} (t_{k} )}} { = [}s_{{b_{ij} (t_{k} )}} {]}_{f \times g}$ are calculated by Eqs. (2) and (3) respectively.
Step 2.: According to expected score matrices $S_{{a_{ij} (t_{k} )}} { = [}s_{{a_{ij} (t_{k} )}} {]}_{f \times g}$ and $S_{{b_{ij} (t_{k} )}} { = [}s_{{b_{ij} (t_{k} )}} {]}_{f \times g}$, the step lengths $\gamma_{ij}^{A} (t_{k} )$ and $\gamma_{ij}^{B} (t_{k} )$ of bilateral subjects are calculated at time $t_{k}$ by Eqs. (4) and (5). On this basis, generalized series $r_{{ijj^{\prime}}} (t_{k} )$ and $r_{{ii^{\prime}j}} (t_{k} )$ between bilateral subjects are calculated at time $t_{k}$ by Eqs. (6) and (7).
Step 3.: Relative satisfactions $\alpha_{{_{{ijj^{\prime}}} }}^{ \succ } (t_{k} )$ and $\alpha_{{_{{ijj^{\prime}}} }}^{?} (t_{k} )$ are obtained at time $t_{k}$ by substituting the generalized series $r_{{ijj^{\prime}}} (t_{k} )$ into Eqs. (10) and (12); then the satisfaction mean matrix $\Phi (t_{k} ) = [\alpha_{ij} \left( {t_{k} } \right)]_{f \times g}$ at time $t_{k}$ is established by Eq. (14).
Step 4.: According to the satisfaction mean matrix $\Phi (t_{k} )$, the growth satisfaction mean matrix $\Delta \Phi (t_{k} ) = [\Delta \alpha_{ij} \left( {t_{k} } \right)]_{f \times g}$ at time $t_{k}$ is calculated by Eq. (15).
Step 5.: Relative satisfactions $\beta_{{_{{ii^{\prime}j}} }}^{ \succ } (t_{k} )$ and $\beta_{{_{{ii^{\prime}j}} }}^{?} (t_{k} )$ are obtained at time $t_{k}$ by substituting the generalized series $r_{{ii^{\prime}j}} (t_{k} )$ into Eqs. (10) and (12); then the satisfaction mean matrix $\Psi (t_{k} ) = [\beta_{ij} \left( {t_{k} } \right)]_{f \times g}$ at time $t_{k}$ is established by Eq. (18).
Step 6.: According to the satisfaction mean matrix $\Psi (t_{k} )$, the growth satisfaction mean matrix $\Delta \Psi (t_{k} ) = [\Delta \beta_{ij} \left( {t_{k} } \right)]_{f \times g}$ of side $B$ to side $A$ is calculated at time $t_{k}$ by Eq. (19).
Step 7.: Growth satisfaction weights $\omega_{{t_{k} }}^{\Delta } (t_{k} = 2,...,q)$ of bilateral subjects are obtained by Eq. (20). According to the initial satisfaction matrix $\Phi (t_{1} )$, the growth satisfaction matrix $\Delta \Phi (t_{k} )$ and weights $\omega_{2}^{\Delta } ,...,\omega_{{t_{k} }}^{\Delta } ,...,\omega_{q}^{\Delta }$, the dynamic satisfaction matrix $\mathop{\Phi }\limits^{\leftrightarrow} = [\mathop{\alpha }\limits^{\leftrightarrow} _{ij} ]_{f \times g}$ of side $A$ to side $B$ is established by Eq. (21).
Step 8.: According to the initial satisfaction matrix $\Psi (t_{1} )$, the growth satisfaction matrix $\Delta \Psi (t_{k} )$ and weights $\omega_{2}^{\Delta } ,...,\omega_{{t_{k} }}^{\Delta } ,...,\omega_{q}^{\Delta }$, the dynamic satisfaction matrix $\mathop{\Psi }\limits^{\leftrightarrow} = [\mathop{\beta }\limits^{\leftrightarrow} _{ij} ]_{f \times g}$ of side $B$ to side $A$ is established by Eq. (22).
Step 9.: According to dynamic satisfaction matrices $\mathop{\Phi }\limits^{\leftrightarrow} = [\mathop{\alpha }\limits^{\leftrightarrow} _{ij} ]_{f \times g}$ and $\mathop{\Psi }\limits^{\leftrightarrow} = [\mathop{\beta }\limits^{\leftrightarrow} _{ij} ]_{f \times g}$ and the matching matrix $X = [x_{ij} ]_{f \times g}$, a stable person-position matching model $(M{ - }1)$ considering dynamic satisfactions is established.
Step 10.: Model $(M{ - }1)$ is transformed into Model $(M{ - }2)$ by the linear weighting method, and Model $(M{ - }2)$ is solved by the relevant software to obtain the optimal stable person-position matching scheme.

## Example analysis

To improve the enthusiasm of employees, an enterprise plans to promote some employees by the end of the year, including four promotion positions $(A_{1} ,A_{2} ,A_{3} ,A_{4} )$. Through layer-by-layer screening, six people $(B_{1} ,B_{2} ,B_{3} ,B_{4} ,B_{5} ,B_{6} )$ get the candidacy. To optimize final matching results, the promotion investigation period is one year, which is divided into four quarters. At the end of each quarter, managers of positions make comprehensive evaluations of candidates' work ability, including skills, eloquence and work experience. Candidates also make comprehensive evaluations for the comfort, interpersonal relationship and salary of promotion positions. Furthermore, the hesitant fuzzy evaluation matrix $A(t_{k} ){ = [}a_{ij} (t_{k} ){]}_{4 \times 6}$ given by managers of positions to candidates is shown in Tables 3, 4, 5, 6; the hesitant fuzzy evaluation matrix $B(t_{k} ){ = [}b_{ij} (t_{k} ){]}_{4 \times 6}$ given by candidates to promotion positions is shown in Tables 7, 8, 9, 10.

**Table 3:** Hesitant fuzzy evaluation matrix $A(t_{1} ){ = [}a_{ij} (t_{1} ){]}_{4 \times 6}$ of managers of positions to candidates in the first quarter.

$A(t_{1} )$	$B_{1}$	$B_{2}$	$B_{3}$	$B_{4}$	$B_{5}$	$B_{6}$
$A_{1}$	{45,60,70}	{48,62,66}	{30,76}	{60,66}	{35,40,60}	{50,60,71}
$A_{2}$	{56,60,73}	{45,73}	{48,60,73}	{45,70}	{45,50}	{45,80}
$A_{3}$	{40,56}	{56,77}	{46,77}	{32,55,66}	{41,68,70}	{48,64,75}
$A_{4}$	{38,71}	{46,70}	{45,59,72}	{65,74}	{47,60}	{45,69,75}

**Table 4:** Hesitant fuzzy evaluation matrix $A(t_{2} ){ = [}a_{ij} (t_{2} ){]}_{4 \times 6}$ of managers of positions to candidates in the second quarter.

$A(t_{2} )$	$B_{1}$	$B_{2}$	$B_{3}$	$B_{4}$	$B_{5}$	$B_{6}$
$A_{1}$	{53,66,70}	{58,66,72}	{64,76}	{61,66}	{62,70,73}	{50,60,72}
$A_{2}$	{58,64,73}	{54,72}	{56,60,70}	{60,66}	{52,59,66}	{61,73}
$A_{3}$	{64,65}	{63,69,75}	{68,73}	{59,65,66}	{67,68,70}	{70,75}
$A_{4}$	{55,67}	{56,69}	{55,67,72}	{68,74}	{56,68}	{66,69,75}

**Table 5:** Hesitant fuzzy evaluation matrix $A(t_{3} ){ = [}a_{ij} (t_{3} ){]}_{4 \times 6}$ of managers of positions to candidates in the third quarter.

$A(t_{3} )$	$B_{1}$	$B_{2}$	$B_{3}$	$B_{4}$	$B_{5}$	$B_{6}$
$A_{1}$	{68,74,85}	{70,76,82}	{70,76}	{77,82,86}	{72,80,83}	{70,80,82}
$A_{2}$	{68,74,83}	{74,82}	{66,80,85}	{77,86}	{72,79,86}	{81,83}
$A_{3}$	{71,81}	{69,75}	{75,83}	{69,86}	{77,78,80}	{80,85}
$A_{4}$	{65,67,74}	{66,79}	{65,76,82}	{68,80}	{76,80,88}	{76,79,85}

**Table 6:** Hesitant fuzzy evaluation matrix $A(t_{4} ){ = [}a_{ij} (t_{4} ){]}_{4 \times 6}$ of managers of positions to candidates in the fourth quarter.

$A(t_{4} )$	$B_{1}$	$B_{2}$	$B_{3}$	$B_{4}$	$B_{5}$	$B_{6}$
$A_{1}$	{84,85}	{80,86,92}	{90,96}	{87,96}	{82,87,93}	{90,92}
$A_{2}$	{88,93}	{84,82,92}	{81,86,95}	{87,96}	{82,89,94}	{91,93}
$A_{3}$	{88,94,98}	{79,95}	{85,93,96}	{89,98}	{87,88,90}	{80,85,97}
$A_{4}$	{85,87}	{86,89,91}	{85,86,92}	{78,94,95}	{86,98}	{76,89}

**Table 7:** Hesitant fuzzy evaluation matrix $B(t_{1} ){ = [}b_{ij} (t_{1} ){]}_{4 \times 6}$ of candidates to positions in the first quarter.

$B(t_{1} )$	$B_{1}$	$B_{2}$	$B_{3}$	$B_{4}$	$B_{5}$	$B_{6}$
$A_{1}$	{34,43,60}	{48,52}	{45,47}	{33,34,46}	{35,60}	{24,45,61}
$A_{2}$	{56,60}	{44,53}	{48,60,65}	{35,45,61}	{42,45,50}	{?}
$A_{3}$	{33,40,56}	{56,64}	{24,46,55}	{32,55}	{41,68}	{48,55}
$A_{4}$	{28,41,57}	{36,45,49}	{?}	{35,44}	{45,47,60}	{45,65}

**Table 8:** Hesitant fuzzy evaluation matrix $B(t_{2} ){ = [}b_{ij} (t_{2} ){]}_{4 \times 6}$ of candidates to positions in the second quarter.

$B(t_{2} )$	$B_{1}$	$B_{2}$	$B_{3}$	$B_{4}$	$B_{5}$	$B_{6}$
$A_{1}$	{43,47,59}	{?}	{47,56}	{46,58}	{57,60}	{45,61,64}
$A_{2}$	{52,60,66}	{56,63}	{55,60,65}	{53,61}	{42,48,50}	{45,67}
$A_{3}$	{46,53,61}	{57,64}	{39,44,52}	{40,55,64}	{57,68}	{48,55,64}
$A_{4}$	{?}	{45,49,58}	{44,67}	{54,57,67}	{49,57}	{55,62}

**Table 9:** Hesitant fuzzy evaluation matrix $B(t_{3} ){ = [}b_{ij} (t_{3} ){]}_{4 \times 6}$ of candidates to positions in the third quarter.

$B(t_{3} )$	$B_{1}$	$B_{2}$	$B_{3}$	$B_{4}$	$B_{5}$	$B_{6}$
$A_{1}$	{59,70}	{62,67,74}	{66,74}	{68,70,75}	{60,77}	{61,64,73}
$A_{2}$	{66,71,78}	{63,70}	{68,75,77}	{63,71,78}	{68,70}	{55,67,73}
$A_{3}$	{73,76}	{73,75.79}	{71,77}	{55,74}	{67,68,79}	{58,65,67}
$A_{4}$	{66,68,69}	{68,79}	{64,67}	{67,74}	{?}	{72,75}

**Table 10:** Hesitant fuzzy evaluation matrix $B(t_{4} ){ = [}b_{ij} (t_{4} ){]}_{4 \times 6}$ of candidates to positions in the fourth quarter.

$B(t_{4} )$	$B_{1}$	$B_{2}$	$B_{3}$	$B_{4}$	$B_{5}$	$B_{6}$
$A_{1}$	{73,75,84}	{77,84}	{76,79,88}	{70,85}	{77,87}	{84,93}
$A_{2}$	{81,88}	{76,83,89}	{85,87}	{73,81,88}	{78,90}	{82,90}
$A_{3}$	{76,81,89}	{79,90,92}	{81,87}	{85,94}	{78,89}	{78,85,92}
$A_{4}$	{84,93}	{78,89}	{74,84,87}	{77,84,92}	{78,79,84}	{75,82,87}

### Solution process of hesitant fuzzy dynamic person-position matching decision

To solve the above problem, the decision process of dynamic person-position matching is as follows:

Step 1: According to hesitant fuzzy evaluation matrices $A(t_{1} ){ = [}a_{ij} (t_{1} ){]}_{4 \times 6}$, $A(t_{2} ){ = [}a_{ij} (t_{2} ){]}_{4 \times 6}$, $A(t_{3} ){ = [}a_{ij} (t_{3} ){]}_{4 \times 6}$, $A(t_{4} ){ = [}a_{ij} (t_{4} ){]}_{4 \times 6}$, $B(t_{1} ){ = [}b_{ij} (t_{1} ){]}_{4 \times 6}$, $B(t_{2} ){ = [}b_{ij} (t_{2} ){]}_{4 \times 6}$, $B(t_{3} ){ = [}b_{ij} (t_{3} ){]}_{4 \times 6}$ and $B(t_{4} ){ = [}b_{ij} (t_{4} ){]}_{4 \times 6}$ of bilateral subjects in four quarters, expected score matrices $S_{{a_{ij} (t_{1} )}} { = [}s_{{a_{ij} (t_{1} )}} {]}_{4 \times 6}$, $S_{{a_{ij} (t_{2} )}} { = [}s_{{a_{ij} (t_{2} )}} {]}_{4 \times 6}$, $S_{{a_{ij} (t_{3} )}} { = [}s_{{a_{ij} (t_{3} )}} {]}_{4 \times 6}$, $S_{{a_{ij} (t_{4} )}} { = [}s_{{a_{ij} (t_{4} )}} {]}_{4 \times 6}$, $S_{{b_{ij} (t_{1} )}} { = [}s_{{b_{ij} (t_{1} )}} {]}_{4 \times 6}$, $S_{{b_{ij} (t_{2} )}} { = [}s_{{b_{ij} (t_{2} )}} {]}_{4 \times 6}$, $S_{{b_{ij} (t_{3} )}} { = [}s_{{b_{ij} (t_{3} )}} {]}_{4 \times 6}$ and $S_{{b_{ij} (t_{4} )}} { = [}s_{{b_{ij} (t_{4} )}} {]}_{4 \times 6}$ of bilateral subjects in four quarters are calculated by Eqs. (2) and (3).
Step 2: According to expected score matrices $S_{{a_{ij} (t_{1} )}} { = [}s_{{a_{ij} (t_{1} )}} {]}_{4 \times 6}$, $S_{{a_{ij} (t_{2} )}} { = [}s_{{a_{ij} (t_{2} )}} {]}_{4 \times 6}$, $S_{{a_{ij} (t_{3} )}} { = [}s_{{a_{ij} (t_{3} )}} {]}_{4 \times 6}$, $S_{{a_{ij} (t_{4} )}} { = [}s_{{a_{ij} (t_{4} )}} {]}_{4 \times 6}$, $S_{{b_{ij} (t_{1} )}} { = [}s_{{b_{ij} (t_{1} )}} {]}_{4 \times 6}$, $S_{{b_{ij} (t_{2} )}} { = [}s_{{b_{ij} (t_{2} )}} {]}_{4 \times 6}$, $S_{{b_{ij} (t_{3} )}} { = [}s_{{b_{ij} (t_{3} )}} {]}_{4 \times 6}$ and $S_{{b_{ij} (t_{4} )}} { = [}s_{{b_{ij} (t_{4} )}} {]}_{4 \times 6}$, step lengths $\gamma_{ij}^{A} (t_{k} )(k = 1,2,3,4)$ and $\gamma_{ij}^{B} (t_{k} )(k = 1,2,3,4)$ of bilateral subjects are calculated at time $t_{k} (k = 1,2,3,4)$ by Eqs. (4) and (5). On this basis, generalized series $r_{{ijj^{\prime}}} (t_{k} )(k = 1,2,3,4)$ and $r_{{ii^{\prime}j}} (t_{k} )(k = 1,2,3,4)$ between bilateral subjects are calculated at time $t_{k} (k = 1,2,3,4)$ by Eqs. (6) and (7).
Step 3: Relative satisfactions $\alpha_{{_{{ijj^{\prime}}} }}^{ \succ } (t_{k} )(k = 1,2,3,4)$ and $\alpha_{{_{{ijj^{\prime}}} }}^{?} (t_{k} )(k = 1,2,3,4)$ are obtained at time $t_{k} (k = 1,2,3,4)$ by substituting generalized series $r_{{ijj^{\prime}}} (t_{k} )(k = 1,2,3,4)$ into Eqs. (10) and (12); then satisfaction means of managers of positions to candidates in four quarters is calculated by Eq. (14), and satisfaction mean matrices $\Phi (t_{1} ) = [\alpha_{ij} \left( {t_{1} } \right)]_{4 \times 6}$, $\Phi (t_{2} ) = [\alpha_{ij} \left( {t_{2} } \right)]_{4 \times 6}$, $\Phi (t_{3} ) = [\alpha_{ij} \left( {t_{3} } \right)]_{4 \times 6}$ and $\Phi (t_{4} ) = [\alpha_{ij} \left( {t_{4} } \right)]_{4 \times 6}$ are established, as shown in Tables 11, 12, 13, 14.

**Table 11:** Satisfaction mean matrix $\Phi (t_{1} )$ of managers of positions to candidates in the first quarter $(\theta = 0)$.

$\Phi (t_{1} )$	$B_{1}$	$B_{2}$	$B_{3}$	$B_{4}$	$B_{5}$	$B_{6}$
$A_{1}$	0.423	0.428	0.323	0.499	0.287	0.453
$A_{2}$	0.461	0.403	0.421	0.385	0.291	0.453
$A_{3}$	0.298	0.536	0.445	0.322	0.418	0.458
$A_{4}$	0.339	0.375	0.382	0.558	0.329	0.439

**Table 12:** Satisfaction mean matrix $\Phi (t_{2} )$ of managers of positions to candidates in the second quarter $(\theta = 0)$.

$\Phi (t_{2} )$	$B_{1}$	$B_{2}$	$B_{3}$	$B_{4}$	$B_{5}$	$B_{6}$
$A_{1}$	0.351	0.399	0.563	0.360	0.479	0.312
$A_{2}$	0.435	0.387	0.367	0.387	0.315	0.494
$A_{3}$	0.327	0.418	0.459	0.309	0.402	0.524
$A_{4}$	0.319	0.344	0.387	0.586	0.335	0.544

**Table 13:** Satisfaction mean matrix $\Phi (t_{3} )$ of managers of positions to candidates in the third quarter $(\theta = 0)$.

$\Phi (t_{3} )$	$B_{1}$	$B_{2}$	$B_{3}$	$B_{4}$	$B_{5}$	$B_{6}$
$A_{1}$	0.364	0.371	0.312	0.519	0.421	0.398
$A_{2}$	0.322	0.375	0.356	0.457	0.396	0.472
$A_{3}$	0.363	0.298	0.429	0.393	0.412	0.537
$A_{4}$	0.290	0.349	0.385	0.378	0.611	0.551

**Table 14:** Satisfaction mean matrix $\Phi (t_{4} )$ of managers of positions to candidates in the fourth quarter $(\theta = 0)$.

$\Phi (t_{4} )$	$B_{1}$	$B_{2}$	$B_{3}$	$B_{4}$	$B_{5}$	$B_{6}$
$A_{1}$	0.280	0.323	0.338	0.286	0.376	0.504
$A_{2}$	0.454	0.372	0.478	0.349	0.432	0.417
$A_{3}$	0.378	0.511	0.413	0.731	0.416	0.388
$A_{4}$	0.646	0.394	0.353	0.455	0.337	0.306

#### *Remark 2*

To save space, this paper only enumerates satisfaction mean matrices in four quarters using the missing correlation degree $\theta = 0$.

## .

Step 4: According to satisfaction mean matrices $\Phi (t_{1} ) = [\alpha_{ij} \left( {t_{1} } \right)]_{4 \times 6}$, $\Phi (t_{2} ) = [\alpha_{ij} \left( {t_{2} } \right)]_{4 \times 6}$,$\Phi (t_{3} ) = [\alpha_{ij} \left( {t_{3} } \right)]_{4 \times 6}$ and $\Phi (t_{4} ) = [\alpha_{ij} \left( {t_{4} } \right)]_{4 \times 6}$, growth satisfaction mean matrices $\Delta \Phi (t_{1} ) = [\Delta \alpha_{ij} \left( {t_{1} } \right)]_{4 \times 6}$, $\Delta \Phi (t_{2} ) = [\Delta \alpha_{ij} \left( {t_{2} } \right)]_{4 \times 6}$ and $\Delta \Phi (t_{3} ) = [\Delta \alpha_{ij} \left( {t_{3} } \right)]_{4 \times 6}$ of managers of positions to candidates are calculated by Eq. (15).
Step 5: Relative satisfactions $\beta_{{_{{ii^{\prime}j}} }}^{ \succ } (t_{k} )(k = 1,2,3,4)$ and $\beta_{{_{{ii^{\prime}j}} }}^{?} (t_{k} )(k = 1,2,3,4)$ are obtained at time $t_{k} (k = 1,2,3,4)$ by substituting generalized series $r_{{ii^{\prime}j}} (t_{k} )(k = 1,2,3,4)$ into Eqs. (10) and (12); then satisfaction means of candidates to positions in four quarters is calculated by Eq. (18), and satisfaction mean matrices $\Psi (t_{1} ) = [\beta_{ij} \left( {t_{1} } \right)]_{4 \times 6}$, $\Psi (t_{2} ) = [\beta_{ij} \left( {t_{2} } \right)]_{4 \times 6}$, $\Psi (t_{3} ) = [\beta_{ij} \left( {t_{3} } \right)]_{4 \times 6}$ and $\Psi (t_{4} ) = [\beta_{ij} \left( {t_{4} } \right)]_{4 \times 6}$ are established, as shown in Tables 15, 16, 17, 18.

**Table 15:** Satisfaction mean matrix $\Psi (t_{1} )$ of candidates to positions in the first quarter $(\theta = 0)$.

$\Psi (t_{1} )$	$B_{1}$	$B_{2}$	$B_{3}$	$B_{4}$	$B_{5}$	$B_{6}$
$A_{1}$	0.377	0.391	0.368	0.334	0.360	0.327
$A_{2}$	0.642	0.370	0.543	0.476	0.338	0.375
$A_{3}$	0.342	0.602	0.329	0.414	0.472	0.407
$A_{4}$	0.330	0.308	0.375	0.357	0.405	0.456

**Table 16:** Satisfaction mean matrix $\Psi (t_{2} )$ of candidates to positions in the second quarter $(\theta = 0)$.

$\Psi (t_{2} )$	$B_{1}$	$B_{2}$	$B_{3}$	$B_{4}$	$B_{5}$	$B_{6}$
$A_{1}$	0.343	0.375	0.376	0.344	0.457	0.384
$A_{2}$	0.453	0.419	0.532	0.413	0.295	0.375
$A_{3}$	0.378	0.433	0.299	0.356	0.545	0.371
$A_{4}$	0.375	0.329	0.439	0.453	0.368	0.412

**Table 17:** Satisfaction mean matrix $\Psi (t_{3} )$ of candidates to positions in the third quarter $(\theta = 0)$.

$\Psi (t_{3} )$	$B_{1}$	$B_{2}$	$B_{3}$	$B_{4}$	$B_{5}$	$B_{6}$
$A_{1}$	0.291	0.327	0.382	0.452	0.362	0.376
$A_{2}$	0.472	0.304	0.488	0.441	0.371	0.352
$A_{3}$	0.600	0.593	0.515	0.294	0.419	0.317
$A_{4}$	0.355	0.494	0.287	0.435	0.375	0.688

**Table 18:** Satisfaction mean matrix $\Psi (t_{4} )$ of candidates to positions in the fourth quarter $(\theta = 0)$.

$\Psi (t_{4} )$	$B_{1}$	$B_{2}$	$B_{3}$	$B_{4}$	$B_{5}$	$B_{6}$
$A_{1}$	0.301	0.330	0.528	0.488	0.359	0.468
$A_{2}$	0.428	0.317	0.345	0.461	0.368	0.480
$A_{3}$	0.505	0.322	0.431	0.512	0.350	0.329
$A_{4}$	0.358	0.431	0.391	0.442	0.571	0.289
Step 6: According to satisfaction mean matrices $\Psi (t_{1} ) = [\beta_{ij} \left( {t_{1} } \right)]_{4 \times 6}$, $\Psi (t_{2} ) = [\beta_{ij} \left( {t_{2} } \right)]_{4 \times 6}$, $\Psi (t_{3} ) = [\beta_{ij} \left( {t_{3} } \right)]_{4 \times 6}$ and $\Psi (t_{4} ) = [\beta_{ij} \left( {t_{4} } \right)]_{4 \times 6}$, growth satisfaction mean matrices $\Delta \Psi (t_{1} ) = [\Delta \beta_{ij} \left( {t_{1} } \right)]_{4 \times 6}$, $\Delta \Psi (t_{2} ) = [\Delta \beta_{ij} \left( {t_{2} } \right)]_{4 \times 6}$ and $\Delta \Psi (t_{3} ) = [\Delta \beta_{ij} \left( {t_{3} } \right)]_{4 \times 6}$ of candidates to positions are calculated by Eq. (19).
Step 7: By Eq. (20), weights of growth satisfaction matrices of bilateral subjects in the last three quarters are calculated as: $\omega_{2}^{\Delta } = 0.186$, $\omega_{3}^{\Delta } = 0.307$ and $\omega_{4}^{\Delta } = 0.506$, respectively. By Eq. (21), the dynamic satisfaction matrix $\mathop{\Phi }\limits^{\leftrightarrow} = [\mathop{\alpha }\limits^{\leftrightarrow} _{ij} ]_{4 \times 6}$ is obtained according to the initial satisfaction matrix $\Phi (t_{1} )$ and growth satisfaction matrices $\Delta \Phi (t_{1} )$,$\Delta \Phi (t_{2} )$ and $\Delta \Phi (t_{3} )$, i.e.,
  $$\mathop{\Phi }\limits^{\leftrightarrow} = [\mathop{\alpha }\limits^{\leftrightarrow} _{ij} ]_{4 \times 6} = \left[ {\begin{array}{*{20}c} {0.382} & {0.393} & {0.400} & {0.506} & {0.274} & {0.489} \\ {0.475} & {0.367} & {0.402} & {0.409} & {0.306} & {0.458} \\ {0.386} & {0.490} & {0.440} & {0.406} & {0.387} & {0.369} \\ {0.360} & {0.412} & {0.386} & {0.532} & {0.395} & {0.328} \\ \end{array} } \right]$$
Step 8: The dynamic satisfaction matrix $\mathop{\Psi }\limits^{\leftrightarrow} = [\mathop{\beta }\limits^{\leftrightarrow} _{ij} ]_{4 \times 6}$ is obtained by Eq. (22) according to the initial satisfaction matrix $\Psi (t_{1} )$, growth satisfaction matrices $\Delta \Psi (t_{1} )$, $\Delta \Psi (t_{2} )$ and $\Delta \Psi (t_{3} )$ and weights $\omega_{2}^{\Delta } = 0.186$, $\omega_{3}^{\Delta } = 0.307$ and $\omega_{4}^{\Delta } = 0.506$, i.e.,
  $$\mathop{\Psi }\limits^{\leftrightarrow} = [\mathop{\beta }\limits^{\leftrightarrow} _{ij} ]_{4 \times 6} = \left[ {\begin{array}{*{20}c} {0.349} & {0.372} & {0.349} & {0.285} & {0.356} & {0.399} \\ {0.604} & {0.378} & {0.523} & {0.427} & {0.384} & {0.401} \\ {0.305} & {0.578} & {0.338} & {0.606} & {0.445} & {0.420} \\ {0.480} & {0.312} & {0.373} & {0.379} & {0.381} & {0.339} \\ \end{array} } \right]$$
Step 9: According to dynamic satisfaction matrices $\mathop{\Phi }\limits^{\leftrightarrow} = [\mathop{\alpha }\limits^{\leftrightarrow} _{ij} ]_{4 \times 6}$ and $\mathop{\Psi }\limits^{\leftrightarrow} = [\mathop{\beta }\limits^{\leftrightarrow} _{ij} ]_{4 \times 6}$ and the matching matrix $X = [x_{ij} ]_{4 \times 6}$, a stable person-position matching model $(M{ - }1)$ considering dynamic satisfactions is established.
Step 10: By the linear weighting method, Model $(M{ - }1)$ is transformed into Model $(M{ - }2)$, where the coefficient matrix $Z = [z_{ij} ]_{4 \times 6}$ is expressed as follows:
  $$Z = [z_{ij} ]_{4 \times 6} = \left[ {\begin{array}{*{20}r} \hfill {0.365} & \hfill {0.382} & \hfill {0.374} & \hfill {0.396} & \hfill {0.315} & \hfill {0.444} \\ \hfill {0.539} & \hfill {0.373} & \hfill {0.462} & \hfill {0.418} & \hfill {0.345} & \hfill {0.429} \\ \hfill {0.345} & \hfill {0.534} & \hfill {0.389} & \hfill {0.506} & \hfill {0.416} & \hfill {0.395} \\ \hfill {0.420} & \hfill {0.362} & \hfill {0.380} & \hfill {0.456} & \hfill {0.388} & \hfill {0.334} \\ \end{array} } \right]$$

Finally, model $(M{ - }2)$ is solved by lingo11 software, and the optimal stable person-position matching matrix is obtained, as shown in Table 19:

**Table 19:** Optimal stable person-position matching matrix $X^{*} = [x_{ij}^{*} ]_{4 \times 6}$.

$x_{ij}^{*}$	$B_{1}$	$B_{2}$	$B_{3}$	$B_{4}$	$B_{5}$	$B_{6}$
$A_{1}$	0	0	0	1	0	0
$A_{2}$	1	0	0	0	0	0
$A_{3}$	0	1	0	0	0	0
$A_{4}$	0	0	0	0	1	0

From Table 19, the optimal stable person-position matching scheme is $\{ (A_{1} ,B_{4} ),(A_{2} ,B_{1} ),(A_{3} ,B_{2} ),(A_{4} ,B_{5} )\}$, and the unmatched scheme is $\{ (B_{3} ,B_{3} ),(B_{6} ,B_{6} )\}$.

### Sensitivity analysis

To illustrate the effectiveness of the method proposed in this paper, the person-position matching problem will be solved from many aspects, and obtained optimal matching schemes are compared. (1) In the case of different correlation parameters, person-position matching models without considering stability constraints are similarly established, and person-position matching schemes in various situations are obtained by solving models, as shown in Table 20. (2) In the case of different correlation parameters $\theta$, person-position matching models considering stability constraints are established, and stable person-position matching schemes in various situations are obtained by solving models, as shown in Table 21. (3) In the case of different weights $\omega_{1}$ and $\omega_{2}$, person-position matching models considering stability constraints are established, and stable person-position matching schemes in various situations are obtained by solving models, as shown in Table 22.

**Table 20:** Person-position matching schemes without considering stability constraints under different correlation parameters $(\omega_{1} = \omega_{2} = 0.5)$.

Correlation parameter $\theta$	Person-position matching scheme	Unmatched scheme
$\theta = 0$	$\{ (A_{1} ,B_{6} ),(A_{2} ,B_{1} ),(A_{3} ,B_{2} ),(A_{4} ,B_{4} )\}$	$\{ (B_{3} ,B_{3} ),(B_{5} ,B_{5} )\}$
$\theta = 0.3$	$\{ (A_{1} ,B_{6} ),(A_{2} ,B_{1} ),(A_{3} ,B_{2} ),(A_{4} ,B_{4} )\}$	$\{ (B_{3} ,B_{3} ),(B_{5} ,B_{5} )\}$
$\theta = 0.5$	$\{ (A_{1} ,B_{6} ),(A_{2} ,B_{1} ),(A_{3} ,B_{2} ),(A_{4} ,B_{4} )\}$	$\{ (B_{3} ,B_{3} ),(B_{5} ,B_{5} )\}$
$\theta = 0.7$	$\{ (A_{1} ,B_{6} ),(A_{2} ,B_{1} ),(A_{3} ,B_{2} ),(A_{4} ,B_{4} )\}$	$\{ (B_{3} ,B_{3} ),(B_{5} ,B_{5} )\}$
$\theta = 1$	$\{ (A_{1} ,B_{6} ),(A_{2} ,B_{1} ),(A_{3} ,B_{2} ),(A_{4} ,B_{4} )\}$	$\{ (B_{3} ,B_{3} ),(B_{5} ,B_{5} )\}$

**Table 21:** Person-position matching schemes considering stability constraints under different correlation parameters $(\omega_{1} = \omega_{2} = 0.5)$.

Correlation parameter $\theta$	Person-position matching scheme	Unmatched scheme
$\theta = 0$	$\{ (A_{1} ,B_{4} ),(A_{2} ,B_{1} ),(A_{3} ,B_{2} ),(A_{4} ,B_{5} )\}$	$\{ (B_{3} ,B_{3} ),(B_{6} ,B_{6} )\}$
$\theta = 0.3$	$\{ (A_{1} ,B_{6} ),(A_{2} ,B_{1} ),(A_{3} ,B_{2} ),(A_{4} ,B_{4} )\}$	$\{ (B_{3} ,B_{3} ),(B_{5} ,B_{5} )\}$
$\theta = 0.5$	$\{ (A_{1} ,B_{6} ),(A_{2} ,B_{1} ),(A_{3} ,B_{2} ),(A_{4} ,B_{4} )\}$	$\{ (B_{3} ,B_{3} ),(B_{5} ,B_{5} )\}$
$\theta = 0.7$	$\{ (A_{1} ,B_{4} ),(A_{2} ,B_{1} ),(A_{3} ,B_{2} ),(A_{4} ,B_{5} )\}$	$\{ (B_{3} ,B_{3} ),(B_{6} ,B_{6} )\}$
$\theta = 1$	$\{ (A_{1} ,B_{4} ),(A_{2} ,B_{1} ),(A_{3} ,B_{2} ),(A_{4} ,B_{5} )\}$	$\{ (B_{3} ,B_{3} ),(B_{6} ,B_{6} )\}$

**Table 22:** Stable person-position matching schemes under different weights $(\theta = 0)$.

Weights $\omega_{1}$ and $\omega_{2}$	Stable person-position matching scheme	Unmatched scheme
$\omega_{1} = 0.1$,$\omega_{2} = 0.9$	$\{(A_{1} ,B_{6} ),(A_{2} ,B_{1} ),(A_{3} ,B_{2} ),(A_{4} ,B_{4} )\}$	$\{ (B_{3} ,B_{3} ),(B_{5} ,B_{5} )\}$
$\omega_{1} = 0.2$,$\omega_{2} = 0.8$	$\{(A_{1} ,B_{4} ),(A_{2} ,B_{1} ),(A_{3} ,B_{2} ),(A_{4} ,B_{5} )\}$	$\{ (B_{3} ,B_{3} ),(B_{6} ,B_{6} )\}$
$\omega_{1} = 0.3$,$\omega_{2} = 0.7$	$\{ (A_{1} ,B_{4} ),(A_{2} ,B_{1} ),(A_{3} ,B_{2} ),(A_{4} ,B_{5} )\}$	$\{ (B_{3} ,B_{3} ),(B_{6} ,B_{6} )\}$
$\omega_{1} = \omega_{2} = 0.5$	$\{ (A_{1} ,B_{4} ),(A_{2} ,B_{1} ),(A_{3} ,B_{2} ),(A_{4} ,B_{5} )\}$	$\{ (B_{3} ,B_{3} ),(B_{6} ,B_{6} )\}$
$\omega_{1} = 0.6$,$\omega_{2} = 0.4$	$\{ (A_{1} ,B_{4} ),(A_{2} ,B_{1} ),(A_{3} ,B_{2} ),(A_{4} ,B_{5} )\}$	$\{ (B_{3} ,B_{3} ),(B_{6} ,B_{6} )\}$
$\omega_{1} = 0.8$,$\omega_{2} = 0.2$	$\{ (A_{1} ,B_{4} ),(A_{2} ,B_{1} ),(A_{3} ,B_{2} ),(A_{4} ,B_{5} )\}$	$\{ (B_{3} ,B_{3} ),(B_{6} ,B_{6} )\}$

It can be seen from Tables 20 and 21 that when different correlation parameters $\theta$ are used, there are some differences among optimal person-position matching schemes obtained by whether considering stability constraints or not. For example, when $\theta = 0$, the optimal person-position matching scheme considering stability constraints is $\{(A_{1} ,B_{6} ),(A_{2} ,B_{1} ),(A_{3} ,B_{2} ),(A_{4} ,B_{4} )\}$, and the unmatched scheme is $\{(B_{3} ,B_{3} ),(B_{5} ,B_{5} )\}$. The optimal person-position matching scheme without considering stability constraints is $\{(A_{1} ,B_{4} ),(A_{2} ,B_{1} ),(A_{3} ,B_{2} ),(A_{4} ,B_{5} )\}$, and the unmatched scheme is $\{(B_{3} ,B_{3} ),(B_{6} ,B_{6} )\}$, which shows the necessity of adding stability constraints in the person-position matching process. It can be seen from Table 22 that there are also slight differences in optimal stable person-position matching schemes obtained by different weights. For example, when $\omega_{1} = 0.1$ and $\omega_{2} = 0.9$ are used, the obtained stable person-position matching scheme is $\{(A_{1} ,B_{6} ),(A_{2} ,B_{1} ),(A_{3} ,B_{2} ),(A_{4} ,B_{4} )\}$, and the unmatched scheme is $\{ (B_{3} ,B_{3} ),(B_{5} ,B_{5} )\}$; therefore, when stable person-position matching models considering dynamic satisfactions are solved, weights of bilateral subjects should be fully considered.

### Comparative analysis of different methods

**Figure 2:**
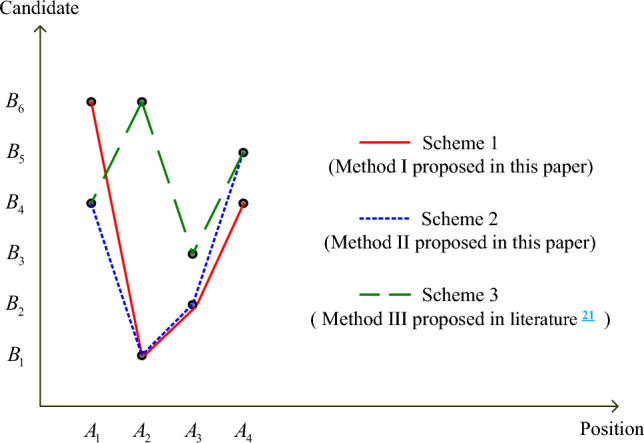
Comparative analysis of optimal matching schemes $(\omega_{1} = \omega_{2} = 0.5)$.

The method proposed in literature^21^ is used to solve the above-mentioned person-position matching problem, and compared with various methods proposed in this paper. Optimal matching schemes obtained by various methods are shown in Fig. 2.

From Fig. 2, it can be seen that optimal person-position matching schemes obtained by two methods proposed in this paper are quite different from that obtained by the method proposed in literature^21^. The optimal matching scheme obtained by Method III proposed in literature^21^ is Scheme 3; while other optimal person-position matching schemes obtained by methods proposed in this paper are Scheme 1 and Scheme 2. Scheme 1 is the person-position matching scheme without considering stability constraints under different correlation parameters $\theta$ and the person-position matching scheme considering stability constraints when correlation parameters are $\theta = 0.3,\theta = 0.5$, that is, $\{ (A_{1} ,B_{6} ),(A_{2} ,B_{1} ),(A_{3} ,B_{2} ),(A_{4} ,B_{4} )\}$; Scheme 2 is the person-position matching scheme considering stability constraints when correlation parameters ($\theta = 0,\theta = 0.7,\theta = 1$) are considered, that is, $\{ (A_{1} ,B_{4} ),(A_{2} ,B_{1} ),(A_{3} ,B_{2} ),(A_{4} ,B_{5} )\}$.

Compared with other methods, advantages of the proposed dynamic person-position matching decision method are as follows: (1) The calculation method for the missing correlation coefficient is proposed, and the correlation parameter $\theta$ is introduced to combine it with the dominant correlation coefficient to calculate relative satisfactions. On this basis, dynamic satisfactions are obtained, which is more convincing. (2) By solving growth satisfactions, we can clearly see changes of satisfaction means in each time period, so that bilateral subjects can make timely improvements to the next stage of behavior. (3) A stable person-position matching model considering dynamic satisfactions is established. The obtained optimal matching scheme is more in line with actual decision needs. Therefore, the optimal person-position matching scheme obtained by methods proposed in this paper is relatively more valuable.

## Conclusions

This paper proposes a dynamic stable person-position matching decision method from the perspective of stable matching under a hesitant fuzzy environment. (1) The missing correlation coefficient is introduced to lay the foundation for the calculation of satisfactions of bilateral subjects at each stage; (2) two correlation coefficients are used to calculate satisfaction means, which can improve the accuracy of satisfactions of bilateral subjects at each stage; (3) the exponential decay formula is used to determine growth satisfaction weights, which is more in line with the actual situation; (4) a stable person-position matching model considering dynamic satisfactions is established, which provides support for solving the person-position matching problem.

Compared with existing methods, main innovations of this paper are as follows: (1) The calculation method for the missing correlation coefficient is proposed. (2) By calculating growth satisfactions, the status of bilateral subjects in each time period is understood, which is convenient for timely improvement of behavior. (3) The calculation method for dynamic satisfactions considering the correlation parameter is proposed. (4) A stable person-position matching model considering dynamic satisfactions is established.

Limitations of this paper are as follows: (1) The considered initial evaluation information is only presented in the form of hesitant fuzzy numbers, without considering other forms of fuzzy preference information. (2) Multi-attribute and multi-group factors in the process of person-position matching are not deeply studied; (3) The dynamic decision method proposed in this paper is difficult to solve the person-position matching problem involving psychological behaviors.

Future research will mainly focus on the following areas: (1) Multiple information expression tools and techniques that are more in line with the real situation need to be used to study person-position matching decision, such as probabilistic hesitant fuzzy sets, hesitant language term sets and test algorithms, etc. (2) Dynamic person-position matching decision under multi-group and multi-attribute conditions can be discussed; (3) The influence of psychological behaviors of bilateral subjects on satisfaction need to be discussed, such as herd mentality, risk aversion behavior and regret behavior. (4) More complex dynamic bilateral matching problems in other fields will be explored.

### Supplementary Information

Supplementary Information.

## Data Availability

The datasets used and/or analysed during the current study available from the corresponding author on reasonable request. The authors confirm that the data supporting the findings of this study are available within the article [and/or its supplementary materials].

## References

[1] Wang TC, Wang XW, Li H (2023). Enhanced prediction accuracy in complex systems: An approach integrating fuzzy K-clustering and fuzzy neural network. Int. J. Knowl. Innov. Stud..

[2] Tešić D, Božanić D, Radovanović M, Petrovski A (2023). Optimising assault boat selection for military operations: An application of the DIBR II-BM-CoCoSo MCDM model. J. Intell. Manag. Decis..

[3] Zhao ZY, Yuan QL (2022). Integrated multi-objective optimization of predictive maintenance and production scheduling: Perspective from lead time constraints. J. Intell. Manag. Decis.

[4] Jana C, Pal M (2023). Interval-valued picture fuzzy uncertain linguistic dombi operators and their application in industrial fund selection. J. Ind Intell..

[5] Riaz M, Farid HMA (2023). Enhancing green supply chain efficiency through linear Diophantine fuzzy soft-max aggregation operators. J. Ind. Intell..

[6] Khan AA, Wang L (2023). Generalized and group-generalized parameter based fermatean fuzzy aggregation operators with application to decision-making. Int. J. Knowl. Innov Stud..

[7] Abid M, Saqlain M (2023). Utilizing edge cloud computing and deep learning for enhanced risk assessment in China’s international trade and investment. Int. J. Knowl. Innov. Stud..

[8] Gale D, Shapley L (1962). College admissions and the stability of marriage. Am. Math. Mon..

[9] Miao Y, Du R, Li J (2019). A two-sided matching model in the context of B2B export cross-border e-commerce. Electron. Commer. Res..

[10] Zhao R, Jin M, Ren P (2020). Stable two-sided satisfied matching for ridesharing system based on preference orders. J. Supercomput..

[11] Han T, Lu J, Zhang H (2023). Two-sided matching model of service providers and demanders considering peer and synergy effects. Heliyon..

[12] Jiang P, Guo S, Du B (2022). Two-sided matching decision-making model for complex product system based on life-cycle sustainability assessment. Expert. Syst. Appl..

[13] Dai W, Hu P (2020). Application of BP neural network in the analytic hierarchy process of person-post evaluation model. J. Supercomput..

[14] Wang X, Jiang Z, Peng L (2021). A deep-learning-inspired person-job matching model based on sentence vectors and subject-term graphs. Complexity..

[15] Beatriz M (2022). Restabilization process in matching markets with workers proposing. Open. J. Disc. Math..

[16] Liu J, Wang S (2021). A method based on TODIM technique for multi-criteria two-sided matching and its application in person-position matching. J. Intell. Fuzzy. Syst..

[17] Yu D, Xu Z (2020). Intuitionistic fuzzy two-sided matching model and its application to personnel-position matching problems. J. Oper. Res. Soc..

[18] Liang Z, Yang Y, Liao S (2022). Interval-valued intuitionistic fuzzy two-sided matching model considering level of automation. Appl. Soft. Comput..

[19] Yang Q, You X, Zhang Y (2021). Two-sided matching based on I-BTM and LSGDM applied to high-level overseas talent and job fit problems. Sci. Rep..

[20] Wang X, Niels A, Alan E (2017). Stable matching for dynamic ride-sharing systems. Transport. Sci..

[21] Zhao X, Zang Y, Luo Y (2018). Method for dynamic two-sided matching decision making based on preference information. Comput. Eng. Appl..

[22] Liang D, He X, Xu Z (2020). Multi-attribute dynamic two-sided matching method of talent sharing market in incomplete preference ordinal environment. Appl. Soft. Comput..

[23] Li H, Shen Q, Bart Y (2021). Dynamic resource allocation on multi-category two-sided platforms. Manag. Sci..

[24] Zhao M, Wang Y, Zhang X, Xu C (2023). Online doctor-patient dynamic stable matching model based on regret theory under incomplete information. Socio-Econ. Plan. Sci..

[25] Chen L, Xu H, Pedrycz W (2023). Conflict analysis based on a novel three-way decisions graph model for conflict resolution method under hesitant fuzzy environment. Inform. Fusion..

[26] Yue Q, Zou W, Hu W (2023). A new theory of triangular intuitionistic fuzzy sets to solve the two-sided matching problem. Alex. Eng. J..

[27] Peng Z, Shan W, Zhu X (2022). Many-to-one stable matching for taxi-sharing service with selfish players. Transport. Res. A. Pol..

[28] Jin L, Gu J, Shu G (1984). Order number methods for MCDM. J. Syst. Sci. Math. Sci..

[29] Slim B, Jean-Marc M (2001). A distance-based collective weak ordering. Group Decis. Negot..

[30] Fernández J, Quintanilla R (2023). Anisotropy can imply exponential decay in micropolar elasticity. Mech. Res. Commun..

