# Supplementary material for: Dynamic person-position matching decision method based on hesitant fuzzy number information
Source: Sci Rep. 2024 Feb 15;14:3846. doi: 10.1038/s41598-024-54177-8 (PMC10869842; doi:10.1038/s41598-024-54177-8)
Supplement: Supplementary file 1 — Supplementary Information. [file 41598_2024_54177_MOESM1_ESM.docx]

**Additional explanation: Data source description**

The supplementary material data comes from the manuscript and is reflected in the form of extended hesitant fuzzy numbers, representing the scoring situation during the matching process between people and positions, with a numerical range of (0-100). This data is aimed at solving the decision problem of person-position matching studied in the manuscript.

The hesitant fuzzy evaluation matrix $A(t_{k})\text{=[}a_{ij}(t_{k})\text{]}_{4\times6}$ given by the managers of positions to candidates is shown in Tables 1-4; the candidate's hesitant fuzzy evaluation matrix $B(t_{k})\text{=[}b_{ij}(t_{k})\text{]}_{4\times6}$ for the promotion positions is shown in Tables 5-8.

| $A(t_{1})$ | $B_{1}$ | $B_{2}$ | $B_{3}$ | $B_{4}$ | $B_{5}$ | $B_{6}$ |
| --- | --- | --- | --- | --- | --- | --- |
| $A_{1}$ | {45,60,70} | {48,62,66} | {30,76} | {60,66} | {35,40,60} | {50,60,71} |
| $A_{2}$ | {56,60,73} | {45,73} | {48,60,73} | {45,70} | {45,50} | {45,80} |
| $A_{3}$ | {40,56} | {56,77} | {46,77} | {32,55,66} | {41,68,70} | {48,64,75} |
| $A_{4}$ | {38,71} | {46,70} | {45,59,72} | {65,74} | {47,60} | {45,69,75} |

**Table 1.** Hesitant fuzzy evaluation matrix $A(t_{1})\text{=[}a_{ij}(t_{1})\text{]}_{4\times6}$ of the managers of positions to candidates in the first quarter.

| $A(t_{2})$ | $B_{1}$ | $B_{2}$ | $B_{3}$ | $B_{4}$ | $B_{5}$ | $B_{6}$ |
| --- | --- | --- | --- | --- | --- | --- |
| $A_{1}$ | {53,66,70} | {58,66,72} | {64,76} | {61,66} | {62,70,73} | {50,60,72} |
| $A_{2}$ | {58,64,73} | {54,72} | {56,60,70} | {60,66} | {52,59,66} | {61,73} |
| $A_{3}$ | {64,65} | {63,69,75} | {68,73} | {59,65,66} | {67,68,70} | {70,75} |
| $A_{4}$ | {55,67} | {56,69} | {55,67,72} | {68,74} | {56,68} | {66,69,75} |

**Table 2.** Hesitant fuzzy evaluation matrix $A(t_{2})\text{=[}a_{ij}(t_{2})\text{]}_{4\times6}$ of the managers of positions to candidates in the second quarter.

| $A(t_{3})$ | $B_{1}$ | $B_{2}$ | $B_{3}$ | $B_{4}$ | $B_{5}$ | $B_{6}$ |
| --- | --- | --- | --- | --- | --- | --- |
| $A_{1}$ | {68,74,85} | {70,76,82} | {70,76} | {77,82,86} | {72,80,83} | {70,80,82} |
| $A_{2}$ | {68,74,83} | {74,82} | {66,80,85} | {77,86} | {72,79,86} | {81,83} |
| $A_{3}$ | {71,81} | {69,75} | {75,83} | {69,86} | {77,78,80} | {80,85} |
| $A_{4}$ | {65,67,74} | {66,79} | {65,76,82} | {68,80} | {76,80,88} | {76,79,85} |

**Table 3.** Hesitant fuzzy evaluation matrix $A(t_{3})\text{=[}a_{ij}(t_{3})\text{]}_{4\times6}$ of the managers of positions to candidates in the third quarter.

| $A(t_{4})$ | $B_{1}$ | $B_{2}$ | $B_{3}$ | $B_{4}$ | $B_{5}$ | $B_{6}$ |
| --- | --- | --- | --- | --- | --- | --- |
| $A_{1}$ | {84,85} | {80,86,92} | {90,96} | {87,96} | {82,87,93} | {90,92} |
| $A_{2}$ | {88,93} | {84,82,92} | {81,86,95} | {87,96} | {82,89,94} | {91,93} |
| $A_{3}$ | {88,94,98} | {79,95} | {85,93,96} | {89,98} | {87,88,90} | {80,85,97} |
| $A_{4}$ | {85,87} | {86,89,91} | {85,86,92} | {78,94,95} | {86,98} | {76,89} |

**Table 4.** Hesitant fuzzy evaluation matrix $A(t_{4})\text{=[}a_{ij}(t_{4})\text{]}_{4\times6}$ of the managers of positions to candidates in the fourth quarter.

| $B(t_{1})$ | $B_{1}$ | $B_{2}$ | $B_{3}$ | $B_{4}$ | $B_{5}$ | $B_{6}$ |
| --- | --- | --- | --- | --- | --- | --- |
| $A_{1}$ | {34,43,60} | {48,52} | {45,47} | {33,34,46} | {35,60} | {24,45,61} |
| $A_{2}$ | {56,60} | {44,53} | {48,60,65} | {35,45,61} | {42,45,50} | {?} |
| $A_{3}$ | {33,40,56} | {56,64} | {24,46,55} | {32,55} | {41,68} | {48,55} |
| $A_{4}$ | {28,41,57} | {36,45,49} | {?} | {35,44} | {45,47,60} | {45,65} |

**Table 5.** Hesitant fuzzy evaluation matrix $B(t_{1})\text{=[}b_{ij}(t_{1})\text{]}_{4\times6}$ of candidates to positions in the first quarter.

| $B(t_{2})$ | $B_{1}$ | $B_{2}$ | $B_{3}$ | $B_{4}$ | $B_{5}$ | $B_{6}$ |
| --- | --- | --- | --- | --- | --- | --- |
| $A_{1}$ | {43,47,59} | {?} | {47,56} | {46,58} | {57,60} | {45,61,64} |
| $A_{2}$ | {52,60,66} | {56,63} | {55,60,65} | {53,61} | {42,48,50} | {45,67} |
| $A_{3}$ | {46,53,61} | {57,64} | {39,44,52} | {40,55,64} | {57,68} | {48,55,64} |
| $A_{4}$ | {?} | {45,49,58} | {44,67} | {54,57,67} | {49,57} | {55,62} |

**Table 6.** Hesitant fuzzy evaluation matrix $B(t_{2})\text{=[}b_{ij}(t_{2})\text{]}_{4\times6}$ of candidates to positions in the second quarter.

| $B(t_{3})$ | $B_{1}$ | $B_{2}$ | $B_{3}$ | $B_{4}$ | $B_{5}$ | $B_{6}$ |
| --- | --- | --- | --- | --- | --- | --- |
| $A_{1}$ | {59,70} | {62,67,74} | {66,74} | {68,70,75} | {60,77} | {61,64,73} |
| $A_{2}$ | {66,71,78} | {63,70} | {68,75,77} | {63,71,78} | {68,70} | {55,67,73} |
| $A_{3}$ | {73,76} | {73,75.79} | {71,77} | {55,74} | {67,68,79} | {58,65,67} |
| $A_{4}$ | {66,68,69} | {68,79} | {64,67} | {67,74} | {?} | {72,75} |

**Table 7.** Hesitant fuzzy evaluation matrix $B(t_{3})\text{=[}b_{ij}(t_{3})\text{]}_{4\times6}$ of candidates to positions in the third quarter.

| $B(t_{4})$ | $B_{1}$ | $B_{2}$ | $B_{3}$ | $B_{4}$ | $B_{5}$ | $B_{6}$ |
| --- | --- | --- | --- | --- | --- | --- |
| $A_{1}$ | {73,75,84} | {77,84} | {76,79,88} | {70,85} | {77,87} | {84,93} |
| $A_{2}$ | {81,88} | {76,83,89} | {85,87} | {73,81,88} | {78,90} | {82,90} |
| $A_{3}$ | {76,81,89} | {79,90,92} | {81,87} | {85,94} | {78,89} | {78,85,92} |
| $A_{4}$ | {84,93} | {78,89} | {74,84,87} | {77,84,92} | {78,79,84} | {75,82,87} |

**Table 8.** Hesitant fuzzy evaluation matrix $B(t_{4})\text{=[}b_{ij}(t_{4})\text{]}_{4\times6}$ of candidates to positions in the fourth quarter.
